# Aberrant miR-339-5p/neuronatin signaling causes prodromal neuronal calcium dyshomeostasis in mutant presenilin mice

**DOI:** 10.1172/JCI149160

**Published:** 2022-04-15

**Authors:** Hao-Yu Zou, Lin Guo, Bei Zhang, Si Chen, Xin-Rong Wu, Xian-Dong Liu, Xin-Yu Xu, Bin-Yin Li, Shengdi Chen, Nan-Jie Xu, Suya Sun

**Affiliations:** 1 Department of Neurology and Institute of Neurology, Ruijin Hospital,; 2 Research Center of Translational Medicine, Shanghai Children’s Hospital, Department of Anatomy and Physiology, and; 3 Shanghai Key Laboratory of Reproductive Medicine, and; 4 Key Laboratory of Cell Differentiation and Apoptosis of Chinese Ministry of Education, Shanghai Jiao Tong University School of Medicine, Shanghai, China.

**Keywords:** Cell Biology, Neuroscience, Alzheimer disease, Calcium signaling, Neurodegeneration

## Abstract

Mushroom spine loss and calcium dyshomeostasis are early hallmark events of age-related neurodegeneration, such as Alzheimer’s disease (AD), that are connected with neuronal hyperactivity in early pathology of cognitive brain areas. However, it remains elusive how these key events are triggered at the molecular level for the neuronal abnormality that occurs at the initial stage of disease. Here, we identify downregulated miR-339-5p and its upregulated target protein, neuronatin (Nnat), in cortex neurons from the presenilin-1 M146V knockin (*PSEN1-M146V* KI) mouse model of familial AD (FAD). Inhibition of miR-339-5p or overexpression of Nnat recapitulates spine loss and endoplasmic reticulum calcium overload in cortical neurons with the *PSEN1* mutation. Conversely, either overexpression of miR-339-5p or knockdown of Nnat restores spine morphogenesis and calcium homeostasis. We used fiber photometry recording during the object-cognitive process to further demonstrate that the *PSEN1* mutant causes defective habituation in neuronal reaction in the retrosplenial cortex and that this can be rescued by restoring the miR-339-5p/Nnat pathway. Our findings thus reveal crucial roles of the miR-339-5p/Nnat pathway in FAD that may serve as potential diagnostic and therapeutic targets for early pathogenesis.

## Graphical abstract

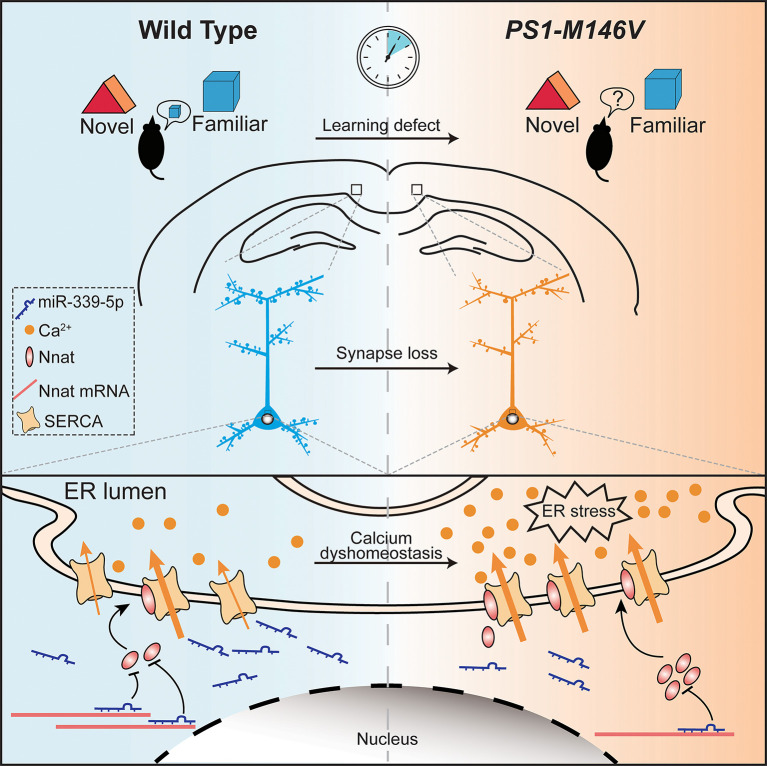

## Introduction

Alzheimer’s disease (AD) is one of the most prevalent age-related neurodegenerative disorders and is characterized by progressive memory impairment and cognitive deficits, often manifested pathologically by the deposition of amyloid-β (Aβ) plaques and neurofibrillary tangles in the brain (1–3). The preclinical phase of AD is designated as mild cognitive impairment (MCI), which is defined as the basis of an abnormal decline of cognition for age (4). Accumulating clinical and experimental evidence has shown that the pathogenic process starts decades before the clinical onset of AD (5, 6). Patients with MCI progress to AD at an annual rate of 5% to 10% compared with 1% to 2% incidence per year among the general population (7). Currently, the most frequently used biomarkers (e.g., Aβ and tau) for AD can only be found using neuroimaging and examination of the cerebrospinal fluid (CSF); these are invasive, expensive, and specialized techniques, which makes them challenging to use in mass screening (5). Recent studies provided strong evidence that microRNAs (miRNAs) could be remarkable and reliable hallmarks owing to their advantages: they are simple and inexpensive to collect and are accurate for use in diagnosis and determining prognosis (8, 9). As early symptoms of AD are less noticeable, an efficient and accessible biomarker that could be found prior to the onset of clinical symptoms would be of critical importance for accurate diagnosis and consistent monitoring during the disease process.

miRNAs are a class of small, noncoding RNAs that participate in posttranscriptional gene expression by binding to the 3′ UTR of target mRNA, resulting in translation repression or degradation (10–12). With a length of about 22 nucleotides, miRNAs exert their work in cell proliferation, neuronal development, synaptic plasticity, and other fundamentally metabolic processes (12, 13). Spatially and temporally specific expression of miRNAs in the brain have suggested a crucial role for miRNAs modulating different physiological and biochemical processes during neurodevelopment. Compelling evidence indicates that perturbation of miRNA expression is associated with the etiology of many human diseases, including neurodegenerative disorders (14–16). Several brain-enriched miRNAs have now been identified that are pathologically altered in AD brains through different signaling pathways, for example, miR-29/β-site amyloid protein precursor (APP) cleaving enzyme 1 (BACE1), miR-132/tau, and miR-124/tyrosine–protein phosphatase nonreceptor type 1 (miR-124/tyrosine-PTPN1) (17–19). Most of the studies identifying these miRNAs are based on the Aβ or tau hypothesis, which aims to alleviate the disease process of advanced stage AD. However, the specific miRNA alteration and the underlying mechanism of pathological events in the prodromal stage of AD are poorly understood.

In this study, we identify a miRNA, miR-339-5p, that is dramatically decreased in brain tissues in an 8-week-old *APP/PS1* transgenic mouse model. The sharp decline of miR-339-5p expression occurs in both the cortex and the hippocampus (HPC) and is also observed in the serum of MCI and AD patients. Furthermore, we find that a neural developmental regulator, neuronatin (Nnat), serves as the target of miR-339-5p and plays a crucial role in regulating calcium homeostasis in the early stages of AD in animals. Thus, our study reveals a miR-339-5p/Nnat pathway that causes synaptic and calcium deficits prior to pathological occurrence, revealing a prodromal molecular marker and a potential therapeutic target for AD.

## Results

### Downregulated miR-339-5p and upregulated target protein Nnat in AD.

**Figure 1:**
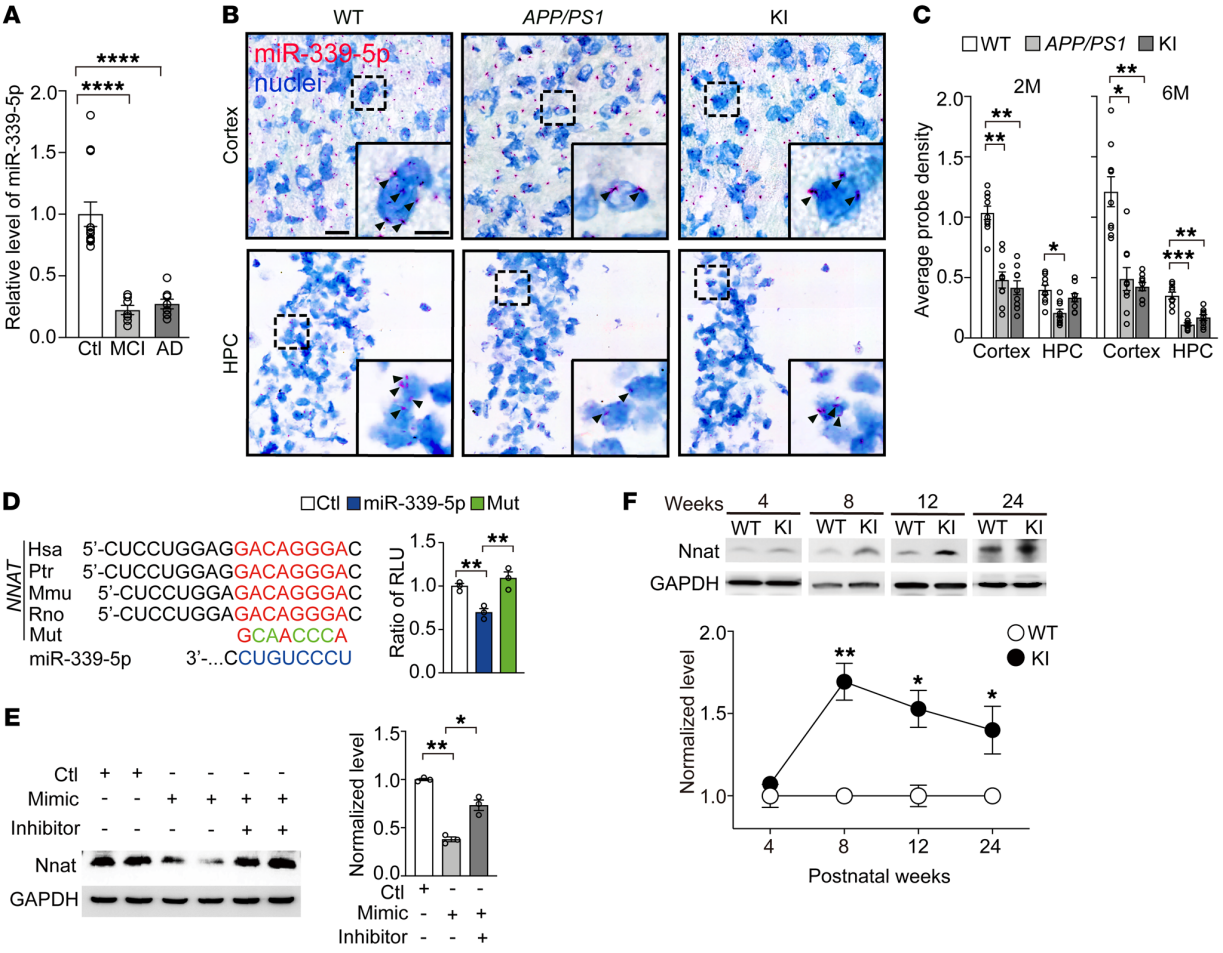
Downregulation of miR-339-5p and elevated Nnat in prodromal AD. (**A**) Expression of miR-339-5p in AD (*n* = 8) and MCI (*n* = 7) patients’ serum compared with age-matched healthy people (*n* = 13). (**B**) Representative images of in situ hybridization staining for miR-339-5p in HPC and cortex on brain slices from 2-month-old WT, *APP/PS1*, and KI mice. Nuclei (blue) were stained with hematoxylin. Black arrows show miR-339-5p–positive dots. Scale bars: 20 μm (left); 10 μm (right). (**C**) Quantification of miR-339-5p probe expression in HPC and cortex for 2-month-old (2M) and 6-month-old transgenic mice. *n* = 9 slices from 3 mice for each group. (**D**) Left: predicted binding sites of miR-339-5p in Nnat 3′ UTR and its mutation (Mut). Right: relative luciferase activity (relative luciferase units [RLU]) was assayed in SH-SY5Y cells transfected with miR-339-5p or scramble control with WT or mutant 3′ UTR of Nnat. *n* = 3 independent experiments. (**E**) Expression levels of Nnat dramatically decreased after treatment with miR-339-5p mimics. *n* = 3 for each group. (**F**) Expression of Nnat was elevated in cortex of KI mice (*n* = 3) compared with age-matched control mice (*n* = 3). Data are represented as mean ± SEM. *P < 0.05; **P < 0.01; ****P < 0.0001. Unpaired *t* test (**F**) and 1-way ANOVA (**A**, and **C**–**E**) were used.

To investigate whether miRNAs are involved in pathological processes in early stage AD, we performed quantitative reverse transcription PCR (qRT-PCR) on total mRNA from brain tissue of 8-week-old *APP/PS1* mice to detect several AD-related miRNAs, as previously reported (20). The result showed that the expression level of miR-339-5p was abnormally lower in *APP/PS1* mice compared with WT littermates (Supplemental Figure 1A; supplemental material available online with this article; https://doi.org/10.1172/JCI149160DS1) and was comparable to the downregulation of miR-16, a verified posttranslational regulator of APP in an AD model (21, 22). We also found miR-339-5p was apparently decreased in serum from either AD or MCI patients (Figure 1A). We further confirmed the distribution of miR-339-5p using in situ hybridization (miRNAScope) and found that miR-339-5p was decreased significantly in the cortex of either *APP/PS1* or presenilin-1 M146V knockin (*PSEN1-M146V* KI) mice at the age of 2 months and 6 months (Figure 1, B and C, and Supplemental Figure 1B). We observed decreased miR-339-5p in HPC in an *APP/PS1* mouse line from postnatal 2 months and in KI mice from postnatal 6 months (Figure 1, B and C, and Supplemental Figure 1B). To further determine whether miR-339-5p downregulation was attributable to Aβ accumulation, we compared endogenous miR-339-5p levels in primary cultured neurons treated with either Aβ conditional or control medium and did not see obvious changes in either WT or KI mice (Supplemental Figure 1C). These results suggest that miR-339-5p would be a specified potential biomarker to represent early biochemical changes in the prodromal stage of AD.

**Figure 2:**
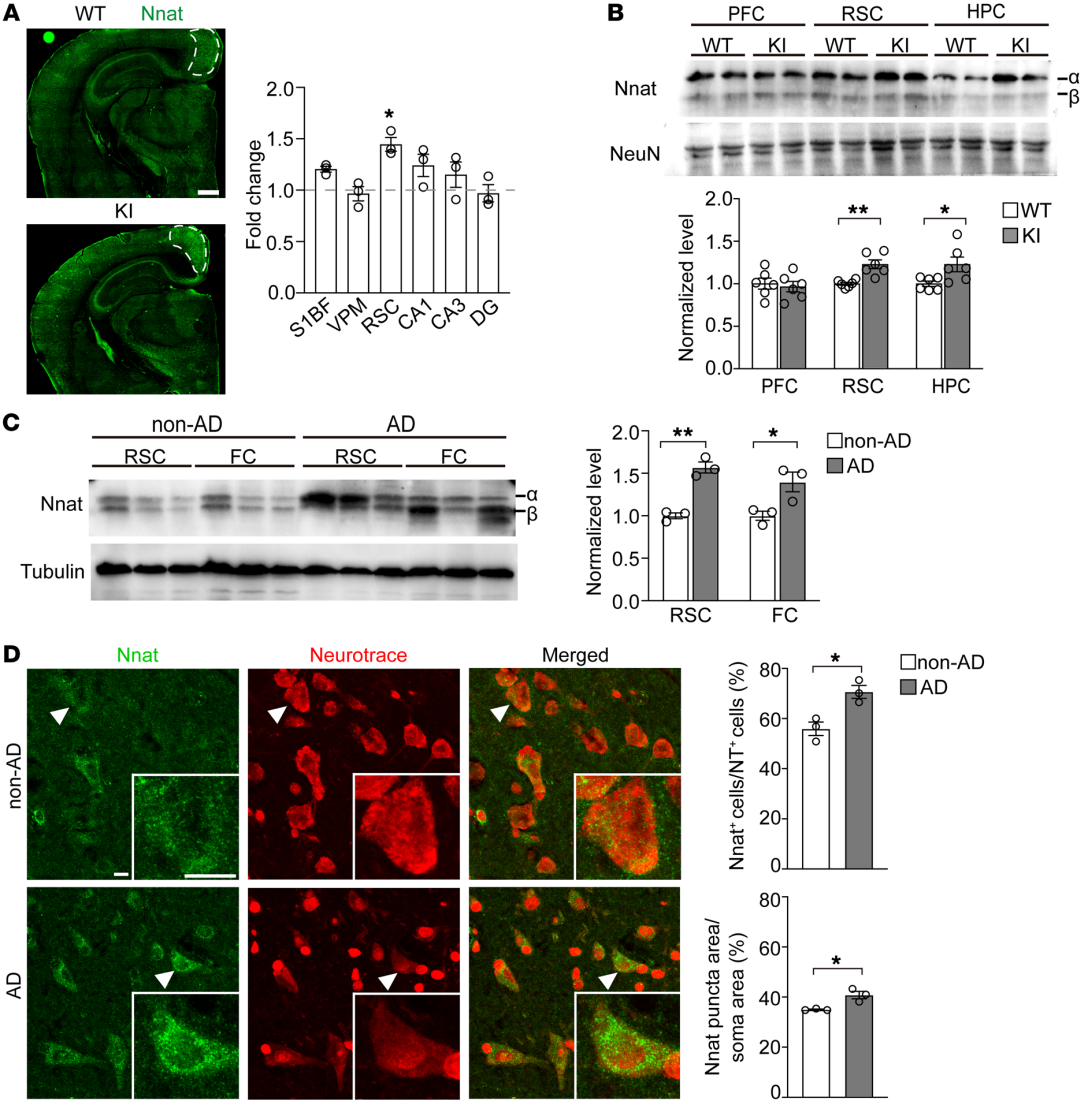
Nnat levels are increased in RSC of AD mice and patients. (**A**) Left: representative images of Nnat distribution by immunostaining in 2-month-old WT and KI mouse brain slices. White dotted lines indicate the RSC region that shows elevated levels of Nnat expression. Scale bar: 500 μm. Right: quantification of fold change of fluorescence (KI/WT) in different brain regions. *n* = 3 mice for each group. S1BF, primary somatosensory cortex, barrel field; VPM, ventral posteromedial thalamic nucleus; CA1, field of CA1 of HPC; CA3, field of CA3 of HPC; DG, dentate gyrus of HPC. (**B**) Nnat levels detected by Western blot in different brain regions of 2- to 3-month-old WT and KI mice. *n* = 6 mice for every group. (**C**) Nnat protein levels were elevated in RSC and FC of AD patients. *n* = 3 subjects for each group. (**D**) Immunostaining of Nnat and Neurotrace showed elevated Nnat^+^ neuron numbers and larger Nnat clusters in RSC of AD patients’ slices compared with control subjects’ slice. *n* = 3 subjects for each group. Arrowheads indicate the representative neurons magnified in lower right parts. Scale bars: 10 μm. All Western blot analysis of Nnat only calculated the α bands. Data are represented as mean ± SEM.*P < 0.05; **P < 0.01. Unpaired *t* test (**B**–**D**) and 2-way ANOVA (**A**) were used.

To predict the target proteins and analyze the function of miR-339-5p, 4 online microRNA target prediction websites, TargetScan (https://www.targetscan.org/vert_80/), PicTar (https://pictar.mdc-berlin.de/), miRDB (http://mirdb.org/), and miRanda (http://www.bioinformatics.com.cn/local_miranda_miRNA_target_prediction_120) databases and Gene Ontology (GO) (http://geneontology.org/) were used. The data suggest that the significantly highlighted potential target genes of miR-339-5p are relative to neurogenesis and synapse development (Supplemental Figure 2, A and B). We screened these targets according to the following artificial restrictive standards: (a) completely matched to binding sites; (b) highly conserved among species; (c) strongly expressed in brain, especially in the cerebral cortex and HPC; and (d) involved in neurodegenerative diseases. Six candidates were selected, including *SLC4A10*, *EPHA4*, *HAP1*, *BCL6*, *BACE1*, and *NNAT*. Western blot analysis showed that Nnat expression was remarkably altered in KI mouse brains compared with other target genes (Supplemental Figure 2D). Accordingly, we found a miR-339-5p decrease was concomitant with an Nnat protein increase, suggesting an involvement of the miR-339-5p/Nnat pathway in the pathogenesis of early stages of the *PSEN1* mutation. To confirm whether miR-339-5p binds directly to Nnat, we transfected firefly luciferase reporter vectors that contained the WT binding site or mutant site within the 3′ UTR of Nnat into SH-SY5Y cells (Figure 1D) and found that expression of miR-339-5p markedly inhibited the activity of the WT luciferase reporter, but not that of seed mutant 3′ UTR (Figure 1D). Furthermore, overexpressed miR-339-5p mimicked reduced Nnat protein levels, while its inhibitor elevated Nnat protein levels (Figure 1E) without interrupting its mRNAs (Supplemental Figure 2C). To describe the temporal profile of the Nnat expression pattern, we performed Western blots for different ages of WT and KI mice and observed a marked increase of Nnat in the cortex from postnatal week 8 (PW8) to PW24 (Figure 1F). We examined expression patterns of Nnat and observed that Nnat was widely distributed in the brain, especially in cognition-related nuclei. As immunofluorescence illustrated, the expression of Nnat in the retrosplenial cortex (RSC), a key brain region that underpins a range of cognitive functions (23–25), in 8-week-old KI mice was significantly higher than that in WT mice (Figure 2A). We further dissected prefrontal cortex (PFC), RSC, and HPC in 2- to 3-month-old WT and KI mice and detected the Nnat protein levels in all these brain areas, observing an increased Nnat level in RSC and HPC of these young KI mice (Figure 2B), which was consistent with the result of immunofluorescence shown in Figure 2A. Moreover, we collected frozen brain tissue from AD patients and non-AD patients and measured the Nnat levels by Western blot analysis. The results showed Nnat levels were increased in either the RSC (Brodmann area 29 and 30) or the frontal cortex (FC) (Figure 2C). We also collected RSC brain slices from AD patients and detected Nnat by immunostaining. As the results showed, Nnat-positive puncta in AD neurons displayed a great deal of clustered distribution and covered a larger area compared with normal brain slices, indicating that Nnat is accumulated in AD (Figure 2D). These results indicate that miR-339-5p directly binds to Nnat mRNA to inhibit its translation, which results in cytoplasmic Nnat accumulation and is involved histopathologically in early stage AD.

### Nnat is associated with ER stress.

Our further analysis showed that Nnat was expressed in neurons of RSC specifically, including both glutamatergic and GABAergic neurons, but not in either astrocytes or microglia (Supplemental Figure 3A). No significant change was observed in KI mice (Supplemental Figure 3A). In line with past findings (26, 27), our analysis revealed that Nnat was localized in ER, but not in mitochondria (Supplemental Figure 3B). We then asked whether Nnat participates in ER dysfunction in the *PSEN1* mutant, which has been shown in previous studies (28–30). To test this hypothesis, we measured protein markers involved in calcium overload and ER stress, including PSD95, BiP/GPR78, and CaMKII, after adding Nnat shRNA in cultured neurons. We found that both BiP/GPR78 and phosphorylated CaMKII (p-CaMKII) were obviously increased in KI cultured neurons, which could be rescued by knocking down Nnat expression (Supplemental Figure 3C). These results indicate that Nnat may be involved in calcium homeostasis in prodromal pathological procedures of the *PSEN1* mutation, which might precede the process of further neurodegeneration.

### Nnat contributes to neural synaptic defects and calcium signal impairment.

**Figure 3:**
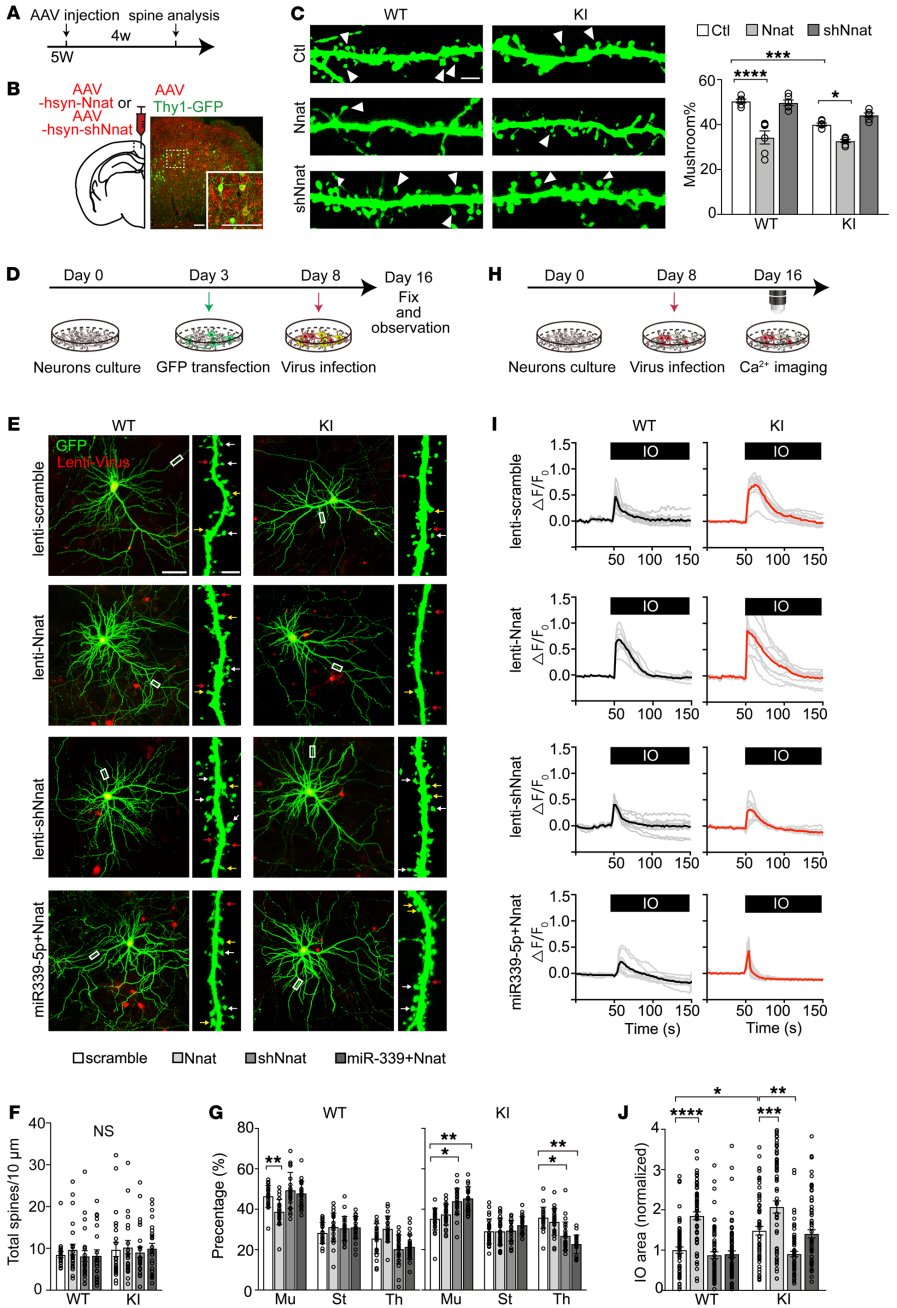
Nnat contributes to neural synaptic and calcium impairments. (**A**) AAV injections were given to 5-week-old (5W) mice, and spine analyses were performed after 4 weeks (4w). (**B**) Left: bilateral injection of AAV into the RSC of Thy1-GFP and M146V; Thy1-GFP mice. Right: representative images of viral expression colocalized with Thy1^+^ neurons (green). Scale bars: 100 μm. (**C**) Left: representative images of spine morphology after viral infection. White arrowheads indicate mushroom-type spines. Scale bar: 2 μm. Right: quantitation of the percentage of mushroom-type spines of different groups. *n* = 6 mice for each group. (**D**) GFP was transfected on DIV3, and lentivirus was added on DIV8. Neurons were ultimately fixed and observed on DIV16. (**E**) Representative cultured neuron images and spine fractions. White arrows indicate mushroom-type spines. Yellow arrows indicate stubby type, and red arrows indicate thin type. Scale bars: 50 μm (left); 2 μm (right). (**F**) Total spine density in cortical neuron cultures; 3 to 5 dendrites per neuron were calculated. *n* = approximately 20–24 neurons for each group. (**G**) Percentages of mushroom (Mu), stubby (St), and thin (Th) spines in cortical neurons cultured from WT and KI mice treated with lentivirus. *n* = approximately 23–32 neurons for each group. (**H**) Lentivirus was added on DIV8, and calcium imaging was performed on DIV16. (**I**) Time courses of Fura-2 Ca^2+^ signals (F340/F380) in the ER of neurons. Individual cell trace (gray) and average trace (black for WT and red for KI) are shown for each group. (**J**) Quantification for average sizes of IO-induced Ca^2+^ estimated as AUC of Fura-2 signal in each group (normalized to control group). *n* = approximately 60–79 neurons for each group. All neurons were analyzed from 3 batches of cultures. Data are represented as mean ± SEM. *P < 0.05; **P < 0.01; ***P < 0.001; ****P < 0.0001, 2-way ANOVA.

Numerous studies have revealed that changes in spine morphogenesis are causally related to calcium regulation by ER in postsynaptic compartments in AD (29, 31). We then asked whether excessive Nnat disrupted spine morphology in vivo. We looked for morphological abnormalities in 4-week-old M146V; Thy1-GFP mice that were treated bilaterally in RSC with AAV2/9 particles containing mouse Nnat complementary DNA or Nnat shRNA (Figure 3, A and B, and Supplemental Figure 4, A and B). The Thy1-GFP transgenic line showed a few excitatory neurons, which allowed us to explore dendritic spine morphology (Figure 3B). The results revealed that, although the total spine density remained unchanged (Supplemental Figure 5A), overexpression of Nnat caused dendrite spines to deteriorate, while inhibition of Nnat rescued mushroom-type spine morphogenesis (Figure 3C and Supplemental Figure 5B).

To further determine the functional interaction of Nnat and miR-339-5p in neurons, we transfected GFP plasmids into WT and KI neurons and examined the morphology of dendritic spines in Nnat and/or miR-339-5p manipulated groups. We infected different groups of neurons on day in vitro 3 (DIV3) by lentivirus containing Nnat or its shRNA vectors, which were labeled by red fluorescence protein, and measured the morphological changes on DIV16 (Figure 3D and Supplemental Figure 4C). Although the total spine density remained unchanged, we observed a significant decrease in populations of mushroom-type spines in WT primary neurons after Nnat overexpression, which was restored to normal levels after additional miR-339-5p expression (Figure 3, E–G), suggesting that dendritic spine maturation is disrupted by excessive Nnat levels. Consistently, we also found obvious reductions in the fraction of mushroom spines by treatment with the miR-339-5p inhibitor in WT neurons, suggesting an essential role of miR-339-5p in maintenance of mature spine morphology (Supplemental Figure 6, A–D). In contrast, we saw an increase in the thin spine population and a decrease in the mushroom spine population in KI primary neurons, which were rescued to a normal level by knocking down Nnat with shRNA (Figure 3, E and G). Likewise, the number of mushroom groups was inversely increased in KI neurons after adding miR-339-5p mimics (Supplemental Figure 6, B and D). These results indicate that aberrant spine structures and numbers in the *PSEN1* mutant neurons are caused by dysregulation of miR-339-5p/Nnat signaling.

Given the accumulating evidence for the disturbance in cellular Ca^2+^ signaling caused by the *PSEN1* mutation (28, 31–33) and Nnat-mediating Ca^2+^ homeostasis (34), we then tested to determine whether miR-339-5p/Nnat affects neuronal morphology and function through regulation of ER-based Ca^2+^ signaling. We used a Fura-2 calcium imaging approach to estimate the total Ca^2+^ store content by eliciting Ca^2+^ efflux from ER to the cytoplasm with ionomycin (IO) treatment (ref. 32 and Figure 3, H and I). We found that Ca^2+^ storage was dramatically increased by overexpression of Nnat in WT neurons that was blocked by a miR-339-5p supplement (Figure 3, I and J). Consistent with previous research (32, 35), KI neurons showed a significant increase in Ca^2+^ content compared with WT neurons, and the excessive rise in cytoplasmic Ca^2+^ concentration was prevented by Nnat knockdown (Figure 3, I and J). In support of the effects of Nnat overexpression, we observed an increase in Ca^2+^ storage in WT neurons after treatment with a miR-339-5p inhibitor (Supplemental Figure 6, E-G). A similar increase of Ca^2+^ content occurred in the KI neurons that could be effectively reduced by exogenous addition of miR-339-5p (Supplemental Figure 6, F and G). The result suggests that the dysregulated miR-339-5p/Nnat pathway shows a direct toxic effect in early neuropathology of *PSEN1* mutant neurons, which inhibits the maturation of dendritic spines and impairs the Ca^2+^-signaling pathway.

### Nnat increases ER calcium storage by facilitating SERCA function.

**Figure 4:**
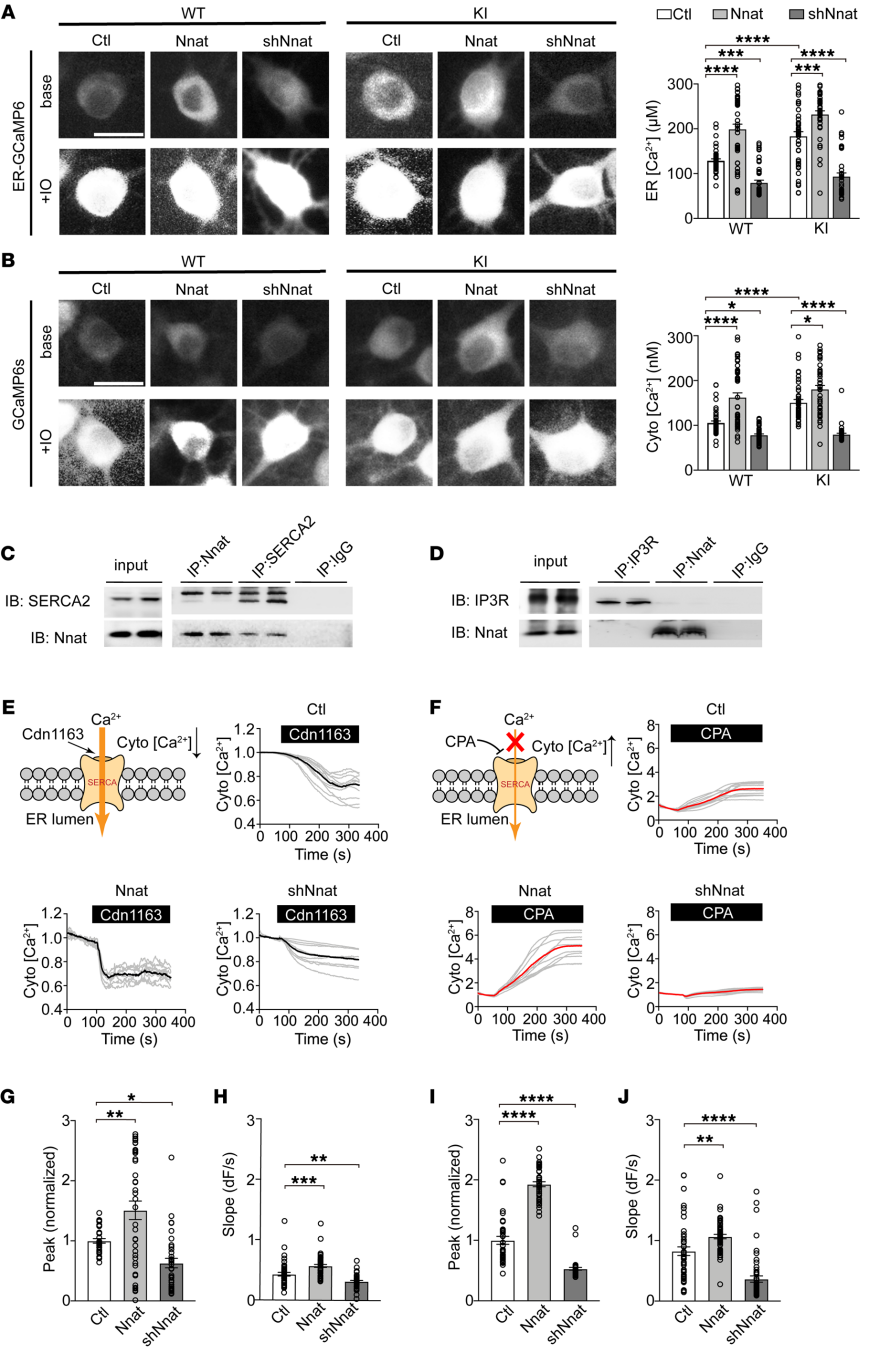
Nnat increases ER calcium storage by facilitating SERCA function. (**A** and **B**) Left: cortical neurons were transfected with ER-GCaMP6 or GCaMP6s to measure ER or cytosolic level. Examples of images before and after application of 50 μM IO. Right: quantification of calcium concentration of ER or cytoplasm at rest. Scale bars: 10 μm. *n* = approximately 42–45 for each group. (**C** and **D**) Coimmunoprecipitation results showed Nnat could interact with SERCA2 but not IP3R. *n* = 3 independent experiments. (**E**) Upper left panel: cdn1163 activated SERCA and promoted Ca^2+^ influx to ER. Upper right and lower panels: representative time courses of cdn1163 Ca^2+^ imaging using GCaMP6s indicator in WT neurons infected with Nnat lentivirus. Individual cell trace (gray) and average trace (black) are shown for each group. (**F**) Upper left panel: CPA inhibited SERCA and promoted Ca^2+^ efflux from ER. Upper right and lower panels: representative time courses of CPA Ca^2+^ imaging using GCaMP6s indicator in WT neurons infected with Nnat lentivirus. Individual cell trace (gray) and average trace are shown for each group. (**G** and **I**) Quantification of peak values normalized to control group of cdn1163 Ca^2+^ imaging and CPA Ca^2+^ imaging. *n* = approximately 32–38 neurons for cdn1163 Ca^2+^ imaging and *n* = approximately 40–43 neurons for CPA Ca^2+^ imaging. (**H** and **J)** Quantification of slope in cdn1163 Ca^2+^ imaging and CPA Ca^2+^ imaging. *n* = approximately 43–51 neurons for cdn1163 Ca^2+^ imaging and *n* = approximately 50–54 neurons for CPA Ca^2+^ imaging. All neurons were analyzed from 4 batches of cultures. Values are shown as mean ± SEM. *P < 0.05; **P < 0.01; ***P < 0.001; ****P < 0.0001. One-way ANOVA (**G**–**J**) and 2-way ANOVA with Tukey’s multiple comparison post hoc test (**A** and **B**) were used.

To clarify how Nnat mediates ER content in neurons, we further investigated the ER calcium content specifically and cytosolic calcium as well using GCaMP6 calcium indicators. We first measured the calcium concentration quantitatively in the ER lumen using ER-GCaMP6-150, an ER calcium indicator (36), and revealed that overexpression of Nnat increased Ca^2+^ of ER (Figure 4A). Consistently with previous Fura-2 results (Figure 3, I and J), overexpression of Nnat in WT neurons elevated ER Ca^2+^ to a high level comparable to that in KI neurons, while knocking down Nnat in KI neurons rescued ER calcium to a level similar to that in WT neurons. Moreover, we also measured the calcium levels in cytoplasm with GCaMP6s, a cytosolic calcium indicator (37), and observed a similar tendency by Nnat overexpression/knocking down in WT and KI neurons (Figure 4B). To further decipher the mechanism of Nnat for calcium regulation, we tested to determine whether Nnat interacted with ER calcium channels sarco/ER Ca^2+^-ATPase (SERCA) and inositol 1,4,5-trisphosphate receptor (IP3R), which mediate calcium transport processes (29), and found that Nnat interacted with SERCA2, but not IP3R (Figure 4, C and D). We thus introduced cdn1163, an activator of SERCA, and cyclopiazonic acid (CPA), a competitive antagonist of SERCA, and measured cytosolic calcium storage by GCaMP6s imaging in WT neurons to clarify the relationship between Nnat and SERCA. We found that cdn1163 induced a decreased cytosolic calcium level in cortical neurons (Figure 4, E–H). The effects were dramatically enhanced in Nnat-overexpressed neurons and were blocked by Nnat shRNA–expressed neurons (Figure 4, E–H). This suggests that Nnat might function to coordinate the activation of SERCA and facilitate Ca^2+^ influx to ER. In contrast, we also observed that SERCA antagonist CPA induced a calcium efflux into cytoplasm (Figure 4F) and the cytosolic Ca^2+^ concentration of highly expressed Nnat neurons increased more rapidly than either control neurons or Nnat shRNA neurons (Figure 4, I and J). This might be accounted for by a larger ER content in Nnat-overexpressed neurons, which could further synchronously increase cytosolic calcium levels. These results indicate that long-term Nnat upregulation could facilitate the activation of SERCA and calcium entry to promote ER storage, which leads to eventual calcium dyshomeostasis in neurons and AD pathological processes.

### The abnormal neuronal activity–associated learning process in RSC of KI mice.

To determine whether *PSEN1-M146V* mutation leads to any behavioral abnormalities, we conducted a battery of behavioral tests for exploratory and learning performance. KI mice showed normal locomotor activity in the open-field test (OFT) and a similar discrimination index in the novel object recognition test (NORT), but with less habituative effect, a behavioral feature for short-term memory during object exploration (Supplemental Figure 7, A and B). We further tested KI mice for spatial memory using the Barnes maze test (BMT) and observed poorer performance during 5-day training trials, though both groups reached the same scores by the end of training (Supplemental Figure 7C). These results suggest that the *PSEN1* mutation causes mild impairment in the learning process rather than long-term memory consolidation and retrieval. Furthermore, no significant difference was found between KI mice and WT mice in social behaviors by the 3-chamber test or dissociative behaviors by the hot-plate test (ref. 38 and Supplemental Figure 7, D and E).

**Figure 5:**
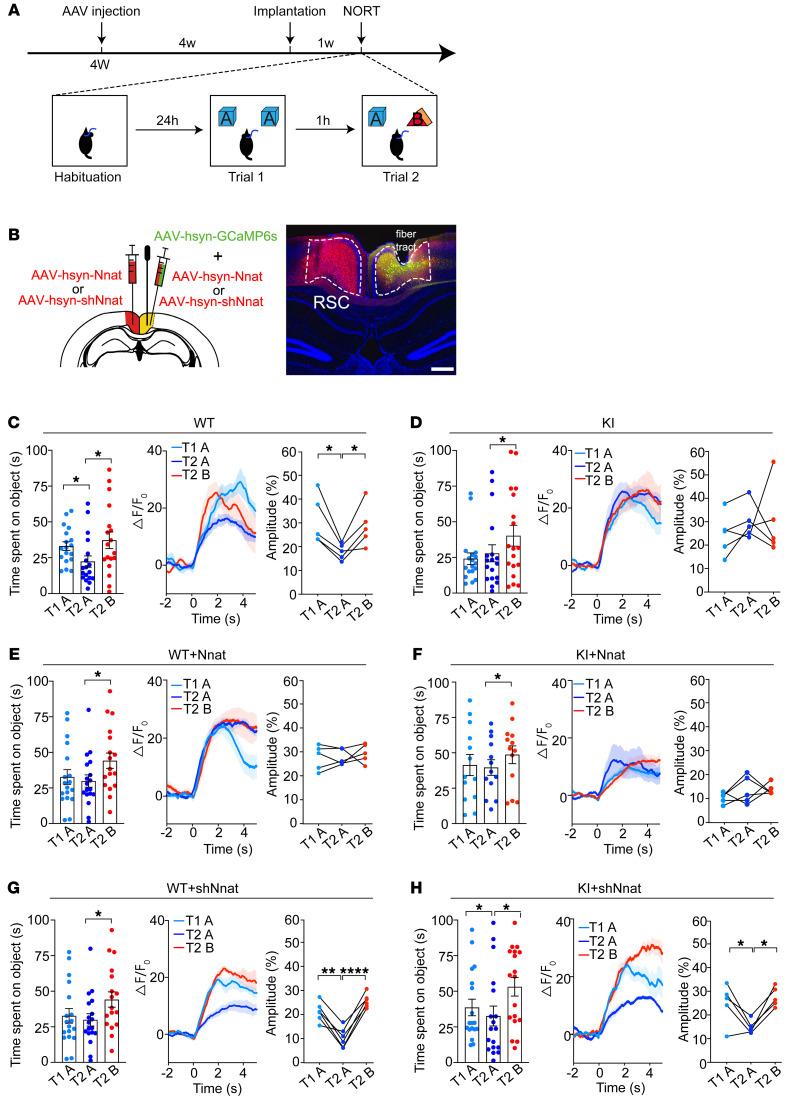
Abnormal neuronal activity is associated with the learning process in RSC of KI mice in short-term NORT. (**A**) Schematic of experimental design. Viral injections were performed on 4-week-old mice, and fibers were implanted after a week. For the NORT, mice were exposed to 2 identical objects (object A) for 10 minutes on trial 1 (T1) after habituation. One hour later, one of the objects in A was replaced by a novel object (object B) for trial 2 (T2). (**B**) Left: schematic of viral injection into the RSC. Right: representative images of viral expression and location of optical fiber tract in RSC. Scale bar: 200 μm. (**C**–**H**) Left: exploring time for different objects. *n* = 18 for each group. Middle: average time courses of Ca^2+^ signal of different objects were shown for each group. Right: amplitudes of peak Ca^2+^ signal changes responding to different objects. (**C** and **D**) WT and KI control mice. *n* = 5 for calcium recording in each group. (**E** and **F**) Nnat-overexpressed WT and KI mice. *n* = 5 for calcium recording in each group. (**G** and **H**) Nnat-knockdown groups. *n* = 6 for calcium recording from WT mice injected with AAV-shNnat. *n* = 5 for calcium recording from KI mice injected with AAV-shNnat. Data are represented as mean ± SEM. *P < 0.05; **P < 0.01; ****P < 0.0001, paired *t* test.

To investigate the role of calcium signal–mediated neuronal activity during the recognitive process, we used fiber photometry; for this, mice were monitored in vivo and allowed to freely explore 2 identical objects (object A) multiple times to determine habituative process (Figure 5, A and B). In addition to obvious behavioral performance in habituation of object recognition in 8-week-old WT mice, we observed a dramatic decline of calcium fluorescence in response to repeated identical object exposures, but not to novel object (object B) exposure (Figure 5C), while KI mice showed a defective habituation and consistently higher calcium signal during object exploration (Figure 5D). Overexpression of Nnat in the RSC region of WT mice resulted in an imitation of abnormal behaviors and calcium signal comparable to that of KI mice (Figure 5, E and F). In contrast, knocking down Nnat showed normal calcium activity and behaviors in WT mice, but restored the behaviors and calcium signal to a normal decline during habituation in KI mice (Figure 5, G and H). To evaluate the effect of viral injection on visual or locomotive abilities of the mice, we conducted a visual behavioral test and OFT and found no significant difference between naive mice and viral-treated mice (Supplemental Figure 7, F and G). These results suggest that Nnat serves as a major mediator for neuronal calcium dyshomeostasis that hereby gives rise to hyperactivity of the RSC region.

**Figure 6:**
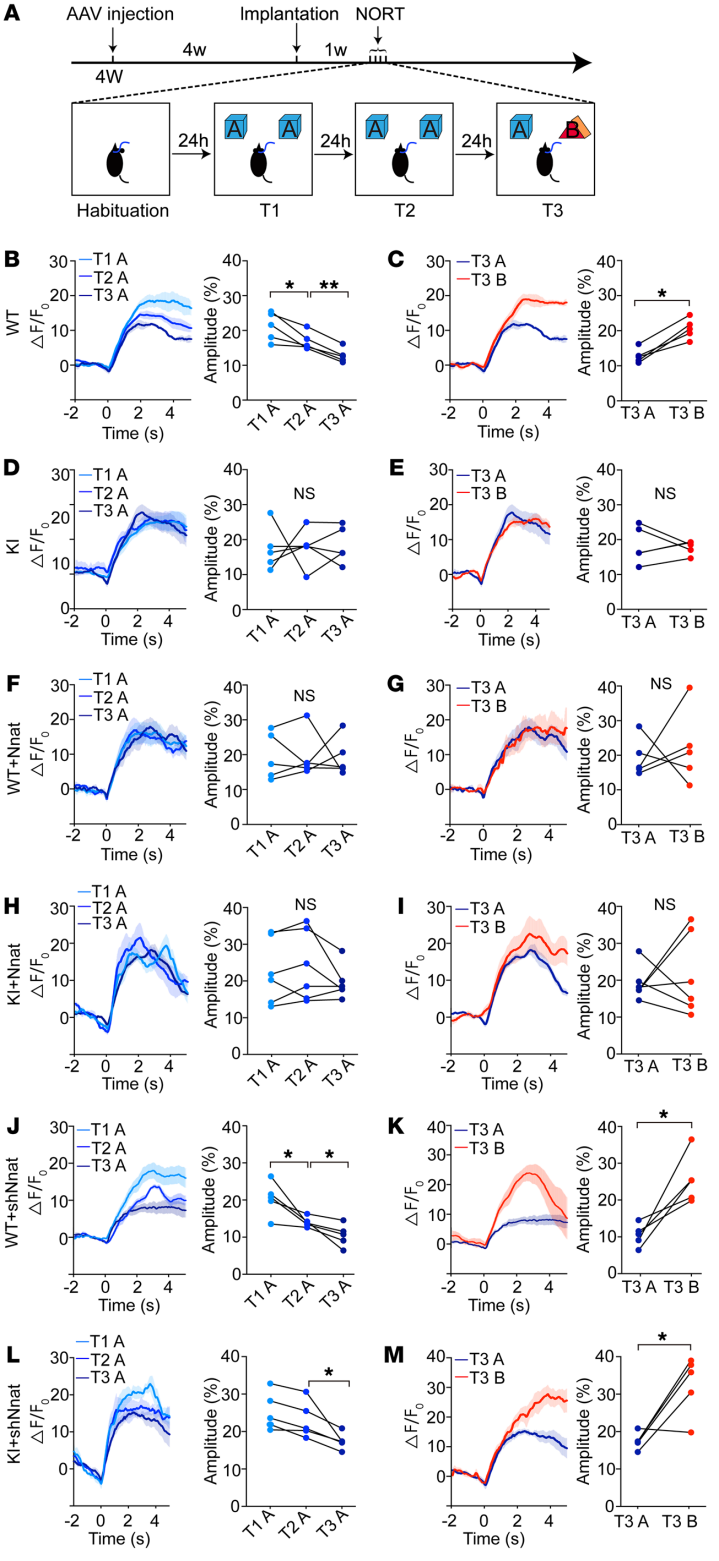
Abnormal neuronal activity is associated with the learning process in RSC of KI mice in long-term NORT. (**A**) Schematic of experimental design. Viral injections were performed on 4-week-old mice, and fibers were implanted after a week. For the NORT, mice were exposed to 2 identical objects (object A) for 10 minutes for 2 consecutive days. On day 3, one of object A was replaced by a novel object (object B) for trial 3 (T3). (**B**–**M**) Ca^2+^ signals recorded from different groups. (**B** and **C**) WT mice treated with control virus showed gradually decreased Ca^2+^ signals for 3 presentations of object A and increased Ca^2+^ signals in response to object B for T3. (**D** and **E**) KI mice treated with control virus showed consistent Ca^2+^ signals for 3 presentations of object A, and on T3, Ca^2+^ signals showed no significant difference when the mice explored familiar or novel objects. (**F** and **G**) Ca^2+^ transients recorded from WT mice treated with Nnat-overexpression virus. (**H** and **I**) Ca^2+^ transients recorded from KI mice treated with Nnat-overexpression virus. (**J** and **K**) Ca^2+^ transients recorded from WT mice treated with Nnat shRNA virus. (**L** and **M**) Ca^2+^ transients recorded from KI mice treated with Nnat shRNA virus. *n* = 6 mice for KI mice injected with AAV-shNnat and *n* = 5 mice for other groups. Values are shown as mean ± SEM. *P < 0.05; **P < 0.01, paired *t* test.

To further investigate whether long-term memory was compromised in KI mice, we performed 2 consecutive day training trials that were followed by novel object discrimination (on day 3) (Figure 6A). We observed that, although exploring time spent by WT control mice on object A showed no significant difference over the 3 repeated presentations of the novelty recognition task (Supplemental Figure 8A), the calcium signals in response to familiar objects gradually declined in contrast with what occurred with the novel object in the RSC calcium recording (Figure 6, B and C). No significant change was seen in KI mice after a 3-day study process (Figure 6, D and E, and Supplemental Figure 8B). These results suggest that calcium dysfunction occurring in RSC may contribute to learning defects, which is in agreement with previous evidence in neuronal hyperactivation as a potential critical feature of early stage AD (39–42). We then tested the roles of Nnat in RSC neuronal hyperactivation and found the Nnat-overexpression groups showed a similar consistent high calcium signal in RSC neurons during the 3-day behavioral process (Figure 6, F–I, and Supplemental Figure 8, A and B), which was comparable to that of KI mice (Figure 6, D and E, and Supplemental Figure 8B). After inhibiting expression of Nnat in RSC of KI mice, we observed a sharp decrease in RSC neuronal calcium responses to familiar objects during the 3-day learning process (Figure 6, L and M, and Supplemental Figure 8B), which is comparable to what occurred with Nnat knockdown in WT mice (Figure 6, J and K, and Supplemental Figure 8A) or WT control mice (Figure 6, B and C, and Supplemental 8A). These results indicate that overloaded Nnat in RSC of KI mice contributes to hyperactive neurons, which is mediated by calcium dyshomeostasis and results in a deficit in both short-term and long-term learning abilities during early stages of AD.

### Inhibition of Nnat rescues behavioral deficits in spatial and object cognition.

**Figure 7:**
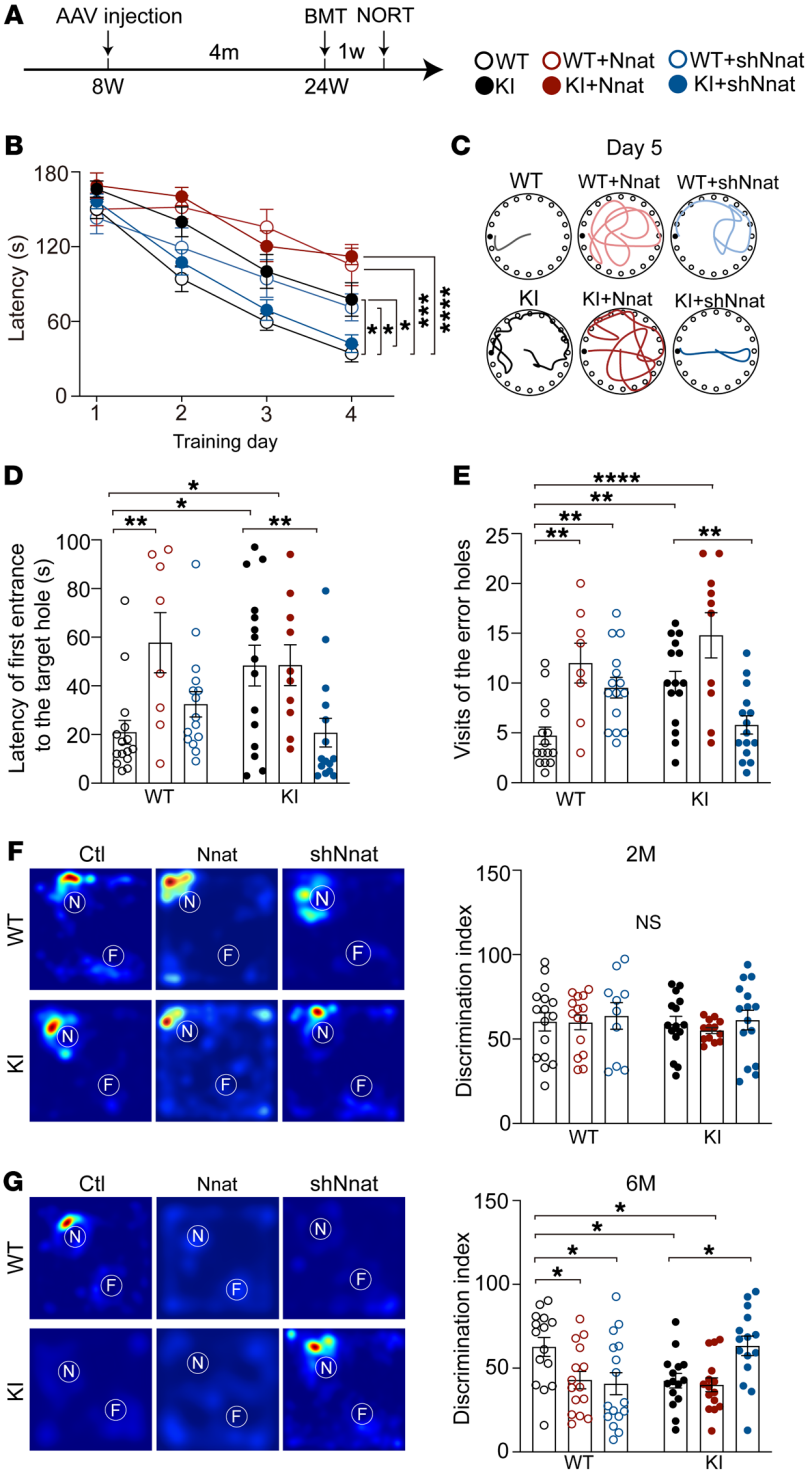
Inhibition of Nnat rescues behavioral deficits in spatial and object cognition. (**A**) Schematic of experimental design. Viral injections were given to 8-week-old mice, and BMTs and NORT (same as Figure 5A) were performed after 4 months. (**B**) For the BMT, latency to the target hole was recorded. *n* = 8 mice for Nnat-overexpressed WT group; *n* = 10 mice for Nnat-overexpressed KI group; *n* = 15 mice for other 4 groups. (**C**) Representative images of exploring paths of different groups on day 5. (**D** and **E**) Latency to the target hole and the number of visits of the error holes on day 5. *n* = 8 mice for Nnat-overexpressed WT group; *n* = 10 mice for Nnat-overexpressed KI group; *n* = 15 mice for other 4 groups. (**F**) Left: representative heatmaps from 6 independent experiments with similar results depicting time of object exploration. White circles represent the location of novel objects (N) and familiar objects (F). Right: 2-month-old mice showed no significant differences in discrimination index in the NORT. *n* = 16 mice for WT control group; *n* = 10 mice for WT mice treated with shNnat; *n* = 13 mice for Nnat-overexpressed KI group; *n* = 15 mice for other 3 groups. For experiment process, also see Figure 5A. (**G**) Left: representative heatmaps from 4 independent experiments depicting time of object exploration. White circles represent the location of novel objects and familiar objects. Right: discrimination index of 6-month-old mice in the NORT. *n* = 16 mice for WT mice treated with shNnat group; *n* = 15 mice for each group. Data are represented as mean ± SEM. *P < 0.05; **P < 0.01; ****P < 0.0001, 2-way ANOVA, Tukey’s test.

Due to the abnormal increase of Nnat in adulthood in KI mice, we hypothesized that interfering with the expression of Nnat in early stage AD could ameliorate behavioral performance in the later stages. Thus, we injected Nnat-related virus into RSC bilaterally at the age of 2 months, tested at the age of 6 months (Figure 7A), and found that KI mice spent a significantly longer time finding the target hole in BMT, while Nnat overexpression in WT mice resulted in a significantly longer latency comparable to that of KI mice (Figure 7B). In contrast, shNnat injection increased latency in WT mice, but reversed prolonged latency to a normal level in KI mice, suggesting a rescued spatial learning ability in shNnat-treated KI mice (Figure 7B). In the probe trials (day 5), we found that both Nnat-overexpressed WT and KI mice exhibited spatial memory deficits at a similar level, as they visited the error holes more often and spent longer times to find the target holes than WT mice (Figure 7, C–E). Furthermore, we found WT mice treated with Nnat shRNA showed slight spatial memory deficits, visiting the error holes more to find the target holes (Figure 7E), whereas KI mice treated with shNnat virus showed a restored normal performance (Figure 7, C–E). Finally we tested these mice for object cognition using NORT. Although no obvious deficit in object cognition was observed at the age of 2 months (Figure 7F), we found that Nnat-overexpressed WT mice imitated poor object recognition at the age of 6 months, while knocking down Nnat chronically in WT mice impaired cognitive ability, resulting in a poor discrimination index, but this knockdown significantly improved the discrimination index in KI mice (Figure 7G). Taken together, these data indicate that Nnat serves as a major cause accounting for the behavioral deficits in spatial and object cognition, which provides a prodromal neuronal marker for AD diagnosis and intervention at the early pathological stage.

## Discussion

The present study uncovers a neural Ca^2+^-signaling pathway, miR-339-5p/Nnat, that mediates abnormal neuronal activity in early stage AD. Among the AD-altered miRNAs at early stages, miR-339-5p is specifically decreased in the cortex, which causes brain abnormalities by targeting Nnat in early pathological conditions. These abnormalities occur in both *APP/PS1* mice, an Aβ-producing model, and *PSEN1-M146V* KI mice, an Aβ-independent familial AD (FAD) model, suggesting a predominate role of the *PSEN1* mutant in prodromal calcium dysfunction in neurons during AD pathogenesis. Several deregulated miRNAs have been documented in the human brain, mouse models, and cell lines, many of which are thought to be involved in the regulation of Aβ generation, synaptic dysfunction, and/or other pathophysiological aspects of AD (12, 13, 16, 20, 43, 44). miR-339-5p has been reported to target BACE1 and downregulate Aβ levels in human brain cultured cells (45). Other studies have profiled miR-339-5p function in cell proliferation by targeting MDM2 or regulating epithelial-to-mesenchymal transition (46, 47). In contrast with these previous studies, our research focuses on incipient AD-associated miRNA targets and identifies Nnat as the target molecule of miR-339-5p that is highly expressed in the brain and upregulated in the early stages of AD. This finding provides a potential biomarker for preclinical diagnosis prior to pathological occurrence of AD.

Nnat is predominately expressed in neonatal brains and declines in adults; it is consider a crucial regulator for influencing neural induction and differentiation (34, 48). Previous studies have shown that Nnat makes contributions to modulation of Ca^2+^ signaling in ER (26, 27). Similarly, the *PSEN1* mutations that cause neurodegeneration in AD probably also act through altered calcium transients that causally precede the development of overt clinical symptoms (29, 49). In the present study, we demonstrate the essential role of Nnat in calcium homeostasis and neuron activity, which are regarded as possibly contributing to the initial pathology of AD (29, 41). Thus, our study provides molecular evidence that the excessive expression of Nnat in KI adult mice might be one of the causes leading to aberrant calcium signaling in the *PSEN1* mutation. Alterations in structures of dendritic spines are believed to be physical substrates for the formation of memory and to be linked with a number of neurological and psychiatric disorders (50–52). As a FAD mice model, *PSEN1-M146V* KI mice have been proven to have deficiency in dendritic spine development and ER calcium release in early stages of AD, which does not rely on Aβ toxicity and tau overphosphorylation. In our research, we showed that these phenotypes could be rescued by replenishment of miR-339-5p or knocking down the abnormally high level of Nnat, which sustains intracellular calcium homeostasis. These results indicate that initial morphological abnormality and neuronal dysfunction during the prodromal stage is mainly dependent on Nnat-mediated calcium storage rather than on Aβ production.

Several lines of evidence, obtained by measuring intracellular Ca^2+^ levels, indicate that Nnat potentially antagonizes the SERCA pump in the ER to block the reuptake of calcium into the ER, resulting in an increase in cytosolic Ca^2+^ (26, 34). In our study, we measured cytosolic calcium content and ER calcium and observed similar elevated cytosolic/ER calcium levels in KI or Nnat-overexpressed WT neurons. Since the calcium levels in both cytoplasm and ER lumen show a similar increasing tendency after Nnat interference, we hypothesized that Nnat might primarily function in regulating ER content levels and that the cytosolic Ca2^+^ level is upregulated, adapting to the overload of ER stores. To validate the role of Nnat in calcium dyshomeostasis, we further regulated intracellular Ca^2+^ by activating or inhibiting the SERCA pump and found that Nnat functions to coordinate the activation of SERCA and facilitate Ca^2+^ influx to the ER. These results indicate that long-term Nnat upregulation might result in larger ER calcium content in neurons and could further synchronously increase cytosolic calcium levels, which is in agreement with our hypothesis. Therefore, our findings suggest that aberrantly increased Nnat contributes to ER calcium overload that may lead to neuronal hyperactivity in *PSEN1* mutant neurons. This shows that a function of Nnat under the *PSEN1* mutation caused ER stress and calcium dyshomeostasis, which compromised spine morphogenesis.

Importantly, Nnat is enriched in RSC, which has been demonstrated to participate in episodic memory, navigation, imagination, and planning for the future (53, 54). The RSC is a mass cortical region that exhibits early signs of neurodegeneration and pathology associated with AD (55, 56) in which heightened excitability of neurons is associated with age-related cognitive decline (57, 58). Our results reveal that neurons in RSCs of KI mice displayed an abnormal consistent hyperactivity during object discrimination, a behavioral deficit in short-term habituation, while knocking down the level of Nnat could rescue the defect. The defects in habituation were also observed in long-term object discrimination in the *PSEN1* mutant, which was also attributable to calcium hyperactivity during processes of memory consolidation or retrieval. Although no obvious memory deficit was observed in these early stages, we speculated that the habituation deficit in neuronal activity might be a prodromal sign for RSC dysfunction that would accelerate AD progression.

In summary, our study demonstrates the crucial role of the miR-339-5p/Nnat pathway in synaptic and calcium dysfunction in early AD, indicating that Nnat could be a potential therapeutic biomarker for early stage AD. Since the diagnosis and therapy of AD are still unsatisfactory, it is urgent and crucial to figure out the prodromal biological and pathological mechanisms for the most prevalent neurodegenerative disease. Our study may lead to a better understanding of how the AD neurons degenerate gradually,which gives rise to eventual cognitive defects, and may provide a pathological target and therapeutic strategy for AD patients.

## Methods

### Animals.

*APP/PS1* mice (APPK670N, M671L; PS1L166P) were provided by Mathias Jucker (Tubingen University). *PSEN1-M146V* KI mice were purchased from the Jackson Laboratory (catalog 004193). Mice were crossed with the *Thy1-GFP-M* transgenic mouse line. Consecutive backcrosses to the S129 strain were performed to move mutations to a S129 background. The ages of mice involved in experiments are described in the text and corresponding figure legends.

### Human serum samples.

Serum of nondementia control participants and MCI and AD patients based on neuropathological diagnosis were collected from patients of the Ruijin Hospital (Shanghai, China). Serum samples were snap-frozen in liquid nitrogen and stored at –80°C until use. Human RSC frozen tissues and RSC brain area paraffin-embedded slices were provided by the Human Brain Bank, Chinese Academy of Medical Sciences, and Peking Union Medical College (Beijing, China). Human FC frozen tissues were provided by the Brain Bank and Neurodegenerative Disease Research Center, University of Science and Technology of China (Anhui, China).

### In situ hybridization.

Animals were anesthetized and perfused with PBS. Fresh-frozen sections from brains of 8-week-old *APP/PS1*, *PSEN1-M146V*, and WT mice were cut into 14 μm thickness and examined using the miRNAscope technique (Advanced Cell Diagnostics). Sections were then fixed in 4% PFA for 15 minutes at 4°C and immersed in ethanol. A few drops of H_2_O_2_ were added to sections for 10 minutes, and sections were washed in PBS, then incubated with Protease III for 30 minutes at room temperature. miR-339-5p probes were preheated to 60°C and added to sections for 2 hours at 40°C in the HybEZ humidified incubator (Advanced Cell Diagnostics), then rinsed in wash buffer and sequentially incubated in reagents AMP1, AMP2, AMP3, AMP4, and AMP5 for 30 minutes. Slides were then immersed in 50% hematoxylin for nuclei staining. Quantification of average probe density is calculated from the number of probe puncta for every 10 μm^2^. Confocal images were than acquired using a Leica SP8 confocal microscope.

### qRT-PCR analysis.

Total RNA from mouse brain tissues was extracted using RNAzol (Thermo Fisher). RNA were then reverse transcribed into cDNA using the TIANScript II RT Kit (Tiangen). The reaction mix was combined with cDNA, SYBER green mix, and corresponding primers. PCR reactions were performed in duplicate for each sample and the average Ct values were used to calculate the mRNA expression levels (CFX96 Touch Real-Time PCR Detection System, Bio-Rad). Ct values of miR-339-5p were normalized to that of U6.

### Western immunoblots.

For Western immunoblots, cortex and HPC samples from *APP/PS1* and *PSEN1-M146V* mice described above were dissected and homogenized in lysis buffer (1% CHAPS, 137 mm NaCl, 2.7 mm KCl, 4.3 mm Na_2_HPO_4_, 1.4 mm KH_2_PO_4_, pH 7.2, 5 mm EDTA, 5 mm EGTA, 1 mm PMSF, 50 mm NaF, 1 mm Na_3_VO_4_, and protease inhibitors) at 4°C for 30 minutes. Lysates were centrifuged at 13,000*g* for 15 minutes at 4°C to remove the insoluble deposit, run on 12% polyacrylamide gels, and then transferred to nitrocellulose membranes. Membranes were blocked in TBST (150 mm NaCl, 10 mm Tris, 0.1% Tween 20, pH 7.6) containing 5% BSA for 1 hour at room temperature. The primary antibodies were diluted in blocking buffer and incubated overnight at 4°C. After washing in TBST, the blots were incubated with HRP-conjugated secondary antibodies for 1 hour at room temperature. After 3 washes, blots were exposed to enhanced chemiluminescence substrate. Quantifications were performed by analyzing relative densities of exposed film using ImageJ (NIH) (RRID:SCR_003070). For primary antibodies, we used rabbit anti-Nnat (1:1000, Abcam, catalog ab27266), mouse anti-SLC4A8/SLC4A10 (1:1000, Invitrogen, catalog PA5-101904), mouse anti-EphA4 (1:1000, Invitrogen, catalog 37-1600), mouse anti-BACE1 (1:1000, Thermo Fisher, catalog MA1-177), rabbit anti-BCL6 (1:1000, Invitrogen, catalog PA5-27390), mouse anti HAP1 (1:1000, Invitrogen, catalog MA1-46412), mouse anti-GAPDH (1:3000, Invitrogen, catalog MA5-15738-D680), rabbit anti-BiP/GRP78 (1:1000, ABclonal, catalog AB2757054), rabbit anti-PSD95 (1:1000, Cell Signaling Technology, catalog 3450), rabbit anti-CaMKII (1:1000, Cell Signaling Technology, catalog 3362), and rabbit anti-phospho-CaMKII (1:1000, Cell Signaling Technology, catalog 12716).

### Luciferase reporter assay.

In our study, we first used TargetScan to acquire the predicted binding site of Nnat mRNA and miR-339-5p. We designed a WT binding site and mutant binding site of 3′ UTR of Nnat and inserted this upstream of the firefly luciferase gene. Then we introduced miR-339-5p plasmid and WT or binding site plasmid into the SHY5Y cell. Twenty-four hours after transfection, luciferase activity was tested.

### Primary cortex neuron culture and dendritic spines analysis.

The cortex cultures of *PSEN1-M146V* KI and WT mice were obtained from P0–P1 pups. Cerebral cortex was isolated and dissociated by 10% (v/v) trypsin (Life Technologies). After digestion for 30 minutes at 37°C, tissues were titrated using a 1 mL fire-polished pipette. For Western immunoblotting, high-density cultures (10^6^ cells per well, plated in 24-well plates) were used, while lower density cultures (2 × 10^5^ cells per well, plated onto glass coverslips in 24-well plates) were used for morphology and immunocytochemical study. All plates and dishes for neural culture were coated with 33 g/mL poly-d-lysin (MilliporeSigma). Cells were cultured in Neurobasal medium (Life Technologies) supplemented with B27 (Life Technologies), 200 Mm glutamine, and 5% FBS. For assessment of synapse morphology, cortical cultures were transfected with GFP plasmid at DIV7 using the calcium phosphate method and fixed (4% formaldehyde, 4% sucrose in PBS, pH 7.4) at DIV16. A *Z*-stack of optical section was captured using a ×100 objective with a confocal microscope (Leica SP8). Quantitative analysis for dendritic spines was performed using the NeuronStudio software package, version 0.9.92 (59). To classify the shape of neuronal spines in culture, we adapted a previously described algorithm (59). For the classification of spine shapes, we used the following cutoff values: aspect ratio for thin spines (AR_thin_(crit)_) = 2.5, head to neck ratio (HNR_(crit)_) = 1.3, and head diameter (HD_(crit)_) = 0.45 μm, where crit indicates critical value. These values were defined and calculated as previously described (57).

### Calcium-imaging experiments.

Cortical cultured neurons were treated with Nnat relative lentivirus or miR-339-5p mimics or inhibitors on DIV8. Calcium-imaging experiments were performed at approximately DIV14–DIV16. For Fura-2 calcium imaging, to measure IO-sensitive calcium pool, neurons were incubated with artificial CSF (aCSF) (140 mM NaCl, 5 mM KCl, 1 mM MgCl_2_, 2 mM CaCl_2_, and 10 mM HEPES, pH 7.3) with Fura-2 (1 μM, Thermo Fisher, F1201) for 30 minutes. After loading, cell glass coverslips were moved to a glass chamber and fixed. Neurons were first immersed in calcium-free aCSF (140 mM NaCl, 5 mM KCl, 1 mM MgCl_2_, 0.4 mM EGTA, and 10 mM HEPES, pH 7.3) for 100 seconds to record the baseline values, and then calcium-free aCSF with IO (1 μM, Beyotime, S1672) was added. The ratios of Fura-2 intensities at excitation wavelengths 340 and 380 nm (R340/380) were taken as measures of the Ca^2+^ pool. The IO area was defined as the area under the IO-induced curve normalized by that of control group.

For cytosolic and ER calcium analysis, GCaMP6s and ER-GCaMP6-150 fluorescence were used to measure the baseline cytosolic Ca^2+^(Cyto [Ca^2+^]) change and Ca^2+^ (ER [Ca^2+^]) change, as previously reported (36, 60). This method relies on measurement of fluorescent indicators at saturating [Ca^2+^](F_max_), which is the peak value obtained by applying aCSF containing 50 μM IO. We first applied aCSF to neurons and measured the baseline (F_0_); then aCSF with 50 μM IO was added and we measured the F_max_. Baseline Cyto[Ca^2+^] was calculated from the following equation: Cyto[Ca^2+^] =Kd[(F_0_/F_max_ – 1/Rf)/(1 – F_0_/F_max_)]^1/n^. As stated in a previous report (37), Kd is the affinity constant (144 nM) of the indicator, Rf (value 98) is the dynamic range, and *n* (2.5) is the Hill coefficient. F_max_ values were not corrected for pH changes. Baseline ER[Ca^2+^] was calculated from the following equation: ER[Ca^2+^] =Kd[(F_0_/F_max_ – 1/Rf)/(1 – F_0_/F_max_)]^1/n^, where Kd is the affinity constant of the ER-GCaMP6-150 indicator (150 mM), F_0_ is the measured fluorescence at rest, Rf is the dynamic range (45), and *n* is the Hill coefficient (1.6; refs. 36, 60).

For cdn1163 and CPA calcium imaging, neurons were treated with Nnat lentivirus on DIV8 and calcium imaging experiments were conducted on DIV16. First, neurons were incubated with aCSF for about 100 seconds, and cdn1163 (50 μM, Selleckchem, S6815) and CPA (5 μM, MedChemExpress, HY-N6771) were dissolved in aCSF and then added to the neurons to mediate ER calcium storage. Analysis of the data was performed using ImageJ software.

### Immunohistochemistry and confocal imaging.

Mice were perfused and brains were fixed with 4% paraformaldehyde (PFA) solution at 4°C. (MilliporeSigma) in PBS. Brain sections were cut at 50 μm thickness coronally. Brain slices were washed in PBS for 10 minutes 3 times and blocked for 1 hour at room temperature with blocking solution: 0.03% Triton X-100 (MilliporeSigma) and 10% donkey serum (Invitrogen) in PBS. Dual-immunofluorescence was performed using the following primary antibodies: rabbit anti-Nnat (1:500, Abcam, catalog ab27266), chicken anti-Tbr1 (1:200, Millipore, catalog AB2261), mouse anti-GAD67 (1:200, Millipore, catalog MAB5406B), mouse anti-parvalbumin (1:500, Millipore, catalog MAB1572), goat anti-somatostatin (1:500, Santa Cruz Biotechnology Inc., catalog sc-7819), goat anti-Iba1 (1:500, Abcam, catalog ab5076), chicken anti-GFAP (1:1000, Millipore, catalog Ab5541), rabbit anti-calnexin (1:1000, Abcam, catalog ab10286), and mouse anti-COX4 (1:500, Abcam, catalog ab110272). Brain slices were incubated with primary antibodies overnight at 4°C in antibody solution: 0.03% Triton X-100 and 2% donkey serum in PBS. Slices were rinsed in PBS for 10 minutes 3 times and then incubated with Alexa Fluor secondary antibodies (Alexa Fluor 488, Alexa Fluor 555, Alexa Fluor 647; 1:500, Thermo Fisher) for 2 hours at room temperature in antibody solution. Finally, slices were rinsed in PBS for 10 minutes 3 times and mounted with DAPI. For paraffin sections of human brains, slides were placed in a 60°C oven for 30 minutes to melt the paraffin before deparaffinization. Slides were deparaffinized in 3 changes of xylene for 10 minutes each. The slides were transferred to 100%, 90%, and 70% alcohol for 10 minutes each and rinsed in PBS for 3 minutes. The slices were incubated with 1× citrate antigen retrieval solution (Beyotime, P0081) at 95°C for 30 minutes and rinsed in PBS for 3 minutes. 10% Donkey serum was applied to brain slices for 2 hours, and rabbit anti-Nnat antibodies (1:100, Thermo Fisher, catalog PA5-115646) were added and incubated overnight at 4°C. Slices were rinsed in PBS for 10 minutes 3 times and then incubated with Alexa Fluor secondary antibodies (Alexa Fluor 488, Alexa Fluor 555, Alexa Fluor 647; 1:500, Thermo Fisher) for 2 hours at room temperature in antibody solution. To block the autofluorescence and eliminate the effects of accumulated lipofuscin, slices were immersed in 0.25% Sudan Black B (A602008, Sangon Biotech) in 70% ethanol for 30 minutes and then rinsed in 70% ethanol until the background was cleaned (61). Finally, slices were rinsed in PBS for 10 minutes 3 times and mounted with DAPI. Confocal images (Leica SP8) were used to obtain high-resolution images of dual-immunofluorescence experiments. Quantitation of colabeled neurons in the cortex was determined with ImageJ software.

### Coimmunoprecipitation.

Mouse brains were dissected and cortex tissues were lysed to obtain protein extracts; 1 μg Nnat antibody (Abcam, catalog ab27266), IP3R antibody (Santa Cruz Biotechnology Inc., catalog sc-377518), or SERCA2 antibody (Invitrogen, catalog MA3-910) was added to protein extracts and reacted overnight at 4°C following incubation with protein G beads for 3 hours at 4°C. Immunoblot experiments were conducted and tested with indicated antibodies.

### Stereotaxic injection and fiber implantation.

Mice were anesthetized using 5% chloral hydrate by intraperitoneal injection. For viral injections in the RSC for spine observation and BMT, the titer of AAV2/9-syn-NNAT-mCherry was 1.13 × 10^12^ VG/mL and 0.9 μl was injected on each side of the RSC. The titer of AAV2/9-syn-shNNAT-mCherry was 2.0 × 10^12^ VG/mL and 0.5 μl was injected on each side of the RSC. The titer of AAV2/9-syn-mCherry was 2.0 × 10^12^ VG/mL and 0.5 μl was injected on each side of the RSC. We bilaterally injected the above virus into the RSC using the following stereotactic coordinates: AP: –2.5 mm, ML: ±0.5 mm, DV: 1.0 mm to bregma skull surface. For novel object–recognition tests and fiber photometry experiments, we not only bilaterally injected Nnat-related virus as previously described, but also injected 500 nL AAV2/9-Syn-GCaMP6s (2.1 × 10^12^ VG/mL) into the right side. Four weeks after injection, a 2.5 mm diameter circular optical fiber was implanted at the coordinates (AP, –2.5 mm; ML, ±0.5 mm; DV, 0.7 mm). Mice were then given 1 week to recover from surgery.

### Fiber photometry.

Customized fiber photometry experiments were performed as previously described (62). Excitation from a 488 nm laser (OBIS 488LS; Coherent) produced fluorescence signals that were reflected by a dichroic mirror (MD498; Thorlabs). The light was transmitted through an optical fiber (200 mm OD, NA = 0.37, 1 m long) between the rotary joint and the implanted fiber. The analog voltage signals were digitized at 50 Hz. The fiber photometry experiment was conducted using a fiber photometry system purchased from ThinkerTech Nanjing BioSicence Inc. For the NORT, mice were allowed to visit objects as described above, along with video recording and fiber photometry acquisition to synchronously record behaviors and calcium signals. All the GCaMP6s signals were subtracted from the background and segmented based on behavioral events in individual trials. We calculated the values of fluorescence change as follows: (ΔF/F) by (F – F_0_)/F_0_, where F_0_ was defined as the average fluorescence signal from 2 seconds preceding event onset. The ΔF/F data were assessed with an average line with a shaded area indicating SEM.

### OFT.

Animals for behavioral tests were randomly selected to experimental groups, and data analysis was performed blinded to the animal genotypes. All animals were habituated to handling in the behavioral room for 3 days prior to the beginning of experiments. All arenas of the animal facility were cleaned with wipes with 75% ethanol to minimize olfactory cues before the next trial. Behavioral tests were performed as previously described (63). For the OFT, mice were exposed to an empty field (40 cm × 40 cm × 40cm) and explored freely for 20 minutes. Locomotor activity was assessed as average velocity.

### NORT.

For the short-term test, mice were habituated for 20 minutes in an arena (40 cm × 40 cm × 40 cm) to minimize anxiety. The next day, mice were allowed to freely visit 2 identical objects for 20 minutes. One hour later, one of the objects was replaced by a novel object that was different in color and shape, but similar in volume. Mice also explored these for 20 minutes. The time spent exploring each object and the discrimination index were calculated as the time spent on novel object divided by the total time spent on novel and familiar objects.

For the long-term test, 24 hours after habituation, mice explored 2 identical objects for 20 minutes for 2 consecutive days. On day 3, mice were exposed to a novel object and a familiar object for 20 minutes. The time spent exploring each object was recorded, and the discrimination index was calculated as the time spent on novel object divided by the total time spent on novel and familiar objects.

### BMT.

The BMT was conducted using a circular platform that is 100 cm in diameter and has 20 evenly spaced holes (4 cm diameter, 2 cm away from the edge). Only one hole led to a drop box in which animals could hide. On the first day, mice were allowed to explore for 3 minutes per trial to find the hiding box. We performed 4 consecutive days of training trials with 4 trials per day. Mice were gently guided to the escape hole if they did not find the hiding box by the end of 3 minutes. On day 5, the hiding box was removed and a 100-second probe trial was performed. Spatial memory abilities were assessed as latency to the hiding box (day 1 to day 4) or the escape hole (day 5).

### Social behavior in 3-chamber test.

To test social behavior in the 3-chamber test, a plexiglass cage was divided into 3 chambers with openings between chambers. The test mice were acclimated to an empty arena for 30 minutes. On the next day, after 5 minute of habituation, mice encountered a never-before-met intruder of the same sex under the perforated cup and an empty perforated cup for 10 minutes. After that, another never-before-met intruder (stranger 2) was placed under the empty cup. The test mouse then freely explored the whole apparatus for 10 minutes. In all these stages, time spent in each chamber and contact with the cup were recorded. To measure social ability, we introduced the sociality index, calculated as the ratio of time spent on stranger 1 to that with the empty cup, and the social novelty index, calculated as the ratio of time spent with stranger 2 to time spent with stranger 1.

### Hot plate test.

Mice were placed on the hot plate (55 °C) and surrounded by a transparent circular container. Mouse activity was recorded by cameras. The experiments ended when mice jumped to escape or 90 seconds had elapsed.

### Elevated plus maze.

The visual function of mice was estimated using light-induced locomotory behavior. We calculated the time it took for the mice to first turn away from a bright flashlight (2 Hz) (60).

### Statistics.

Results are shown as mean ± SEM. For statistical differences, 2-tailed unpaired *t* test or paired *t* test was used for experiments with 2 groups and 1-way ANOVA or 2-way ANOVA followed by Tukey’s post hoc test was used for multiple comparisons in experiments with more than 2 groups.

### Study approval.

Ethical approval for human studies was received from the Ruijin Hospital Ethics Committee (reference number: 2011-10). All patients gave written, informed consent. Mice were used in accordance with the NIH *Guide for the Care and Use of Laboratory Animals* (National Academies Press, 2011) under an IACUC-approved protocol and at an Association for Assessment and Accreditation of Laboratory Animal Care–approved facility at the Shanghai Jiao Tong University School of Medicine.

## Author contributions

SS and NJX conceived and designed the research study. HYZ, LG, and BZ performed the experiments with assistance from Si Chen, XRW, XDL, and XYX. The human serum samples were collected and processed by Shengdi Chen and BYL.

## Supplementary Material

Supplemental data

## Version 1 04/15/2022

Electronic publication
