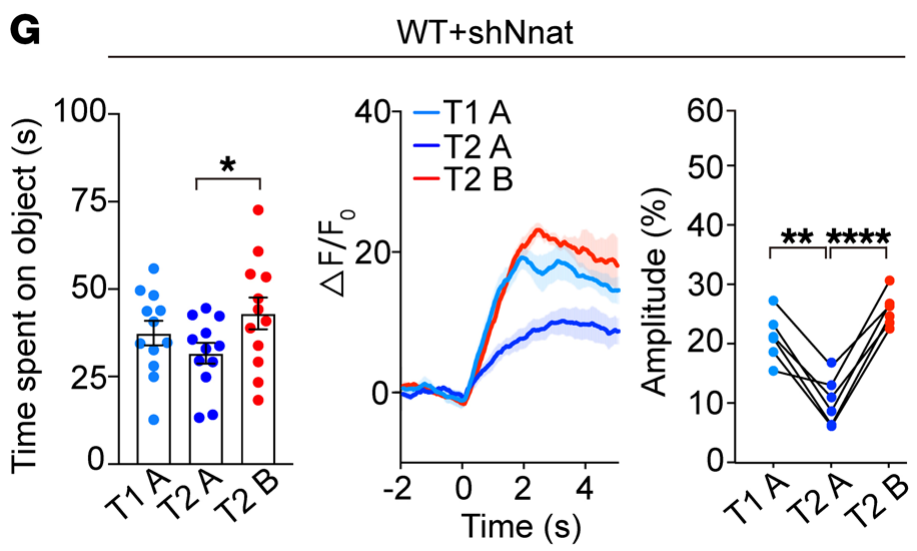# Aberrant miR-339-5p/neuronatin signaling causes prodromal neuronal calcium dyshomeostasis in mutant presenilin mice

**DOI:** 10.1172/JCI168441

**Published:** 2023-02-01

**Authors:** Hao-Yu Zou, Lin Guo, Bei Zhang, Si Chen, Xin-Rong Wu, Xian-Dong Liu, Xin-Yu Xu, Bin-Yin Li, Shengdi Chen, Nan-Jie Xu, Suya Sun

Original citation: *J Clin Invest*. 2022;132(8):e149160. https://doi.org/10.1172/JCI149160

Citation for this corrigendum: *J Clin Invest*. 2023;133(3):e168441. https://doi.org/10.1172/JCI168441

The left panel of Figure 5G originally showed a histogram that was inadvertently duplicated from Figure 5E. The correct figure panel is shown below, and the HTML and PDF versions have been updated online. The correction does not affect the results or conclusions of the article.

The authors regret the error.

## Figures and Tables

**Figure:**