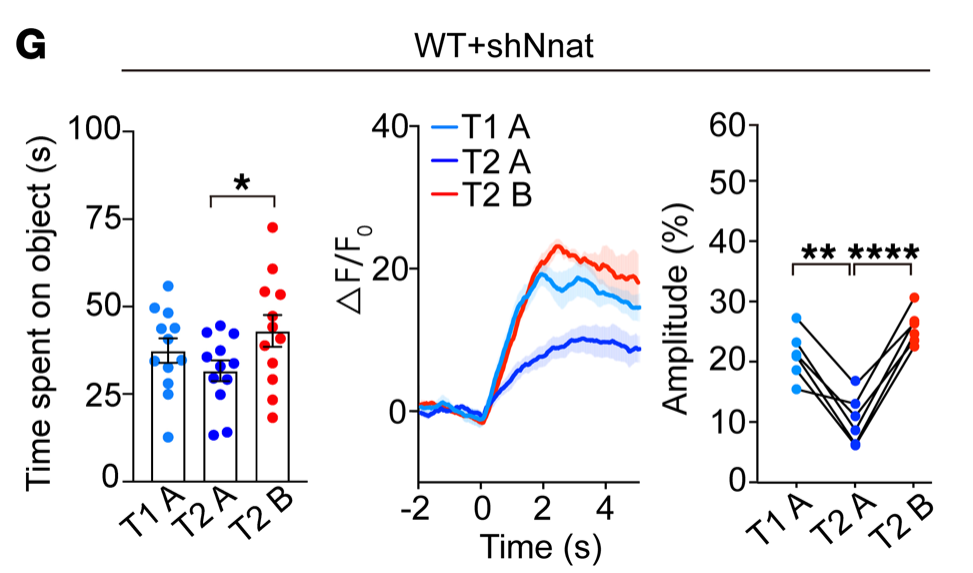# Aberrant miR-339-5p/neuronatin signaling causes prodromal neuronal calcium dyshomeostasis in mutant presenilin mice

**DOI:** 10.1172/JCI169139

**Published:** 2023-02-15

**Authors:** Hao-Yu Zou, Lin Guo, Bei Zhang, Si Chen, Xin-Rong Wu, Xian-Dong Liu, Xin-Yu Xu, Bin-Yin Li, Shengdi Chen, Nan-Jie Xu, Suya Sun

Original citation: *J Clin Invest*. 2022;132(8):e149160. https://doi.org/10.1172/JCI149160

Citation for this corrigendum: *J Clin Invest*. 2023;133(4):e169139. https://doi.org/10.1172/JCI169139

During the preparation of the manuscript, a histogram for Figure 5E was inadvertently pasted into the left panel of Figure 5G. The error does not affect the results or conclusions of the article. The correct figure part is below, and the HTML and PDF versions have been updated online. 

The authors regret the error. 

## Figures and Tables

**Figure:**