# Supplementary material for: Aberrant miR-339-5p/neuronatin signaling causes prodromal neuronal calcium dyshomeostasis in mutant presenilin mice
Source: J Clin Invest. 2022 Apr 15;132(8):e149160. doi: 10.1172/JCI149160 (PMC9012292; doi:10.1172/JCI149160)
Supplement: Supplemental data [file jci-132-149160-s185.pdf]

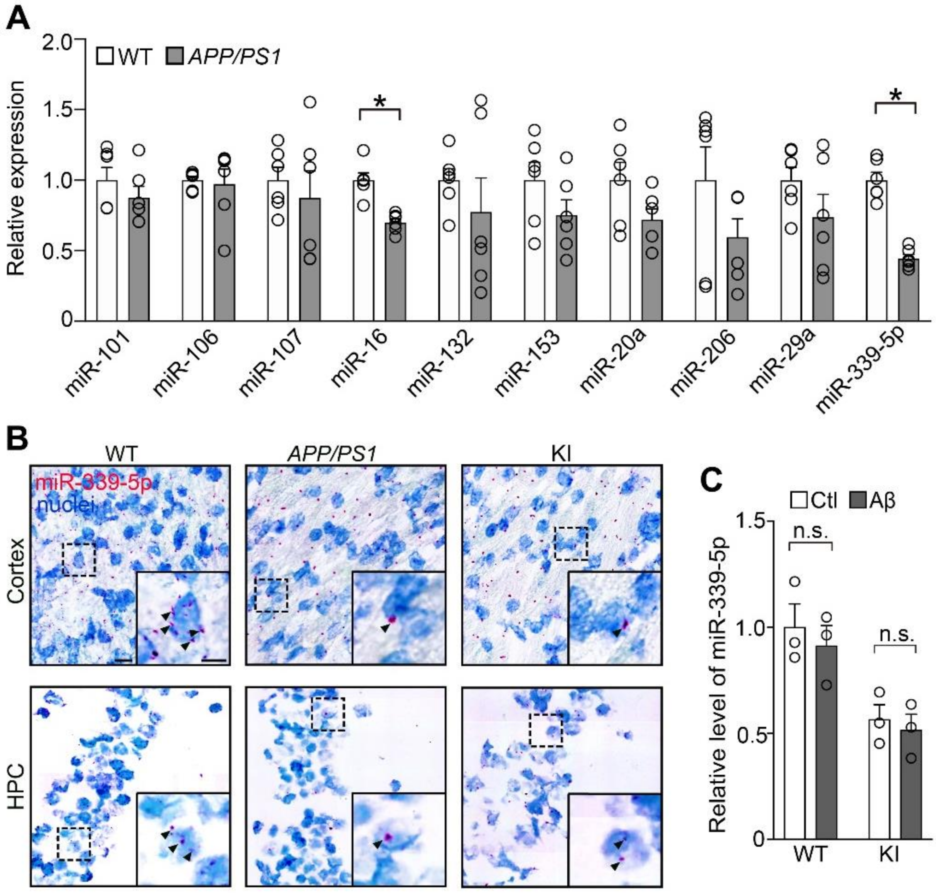

Supplemental Figure 1. Downregulation of miR-339-5p in early stage of AD.

(**A**) Relative expressions of AD-altered miRNAs in brains of 8-week-old *APP/PS1* and WT mice (n=6 mice for each group). (**B**) Representative images of miR-339-5p (red) in hippocampus (HPC) and cortex of WT, *APP/PS1* and KI mice at 6 months. Nuclei (blue) is staining with hematoxylin. Black arrows indicate the positive bots of miR-339-5p. Scale bars: left, 20 μm; right, 10 μm. Quantification data are shown in Figure 1C. (**C**) DIV16 WT and KI hippocampus neurons were treated with 5 μM Aβ42 oligomer or scramble control for 24 hours, and qRT-PCR were performed to test the expression of miR-339-5p, n=3 independent experiments for each group. Data are shown as mean ± SEM. * $p<0.05$. Unpaired t test was used.

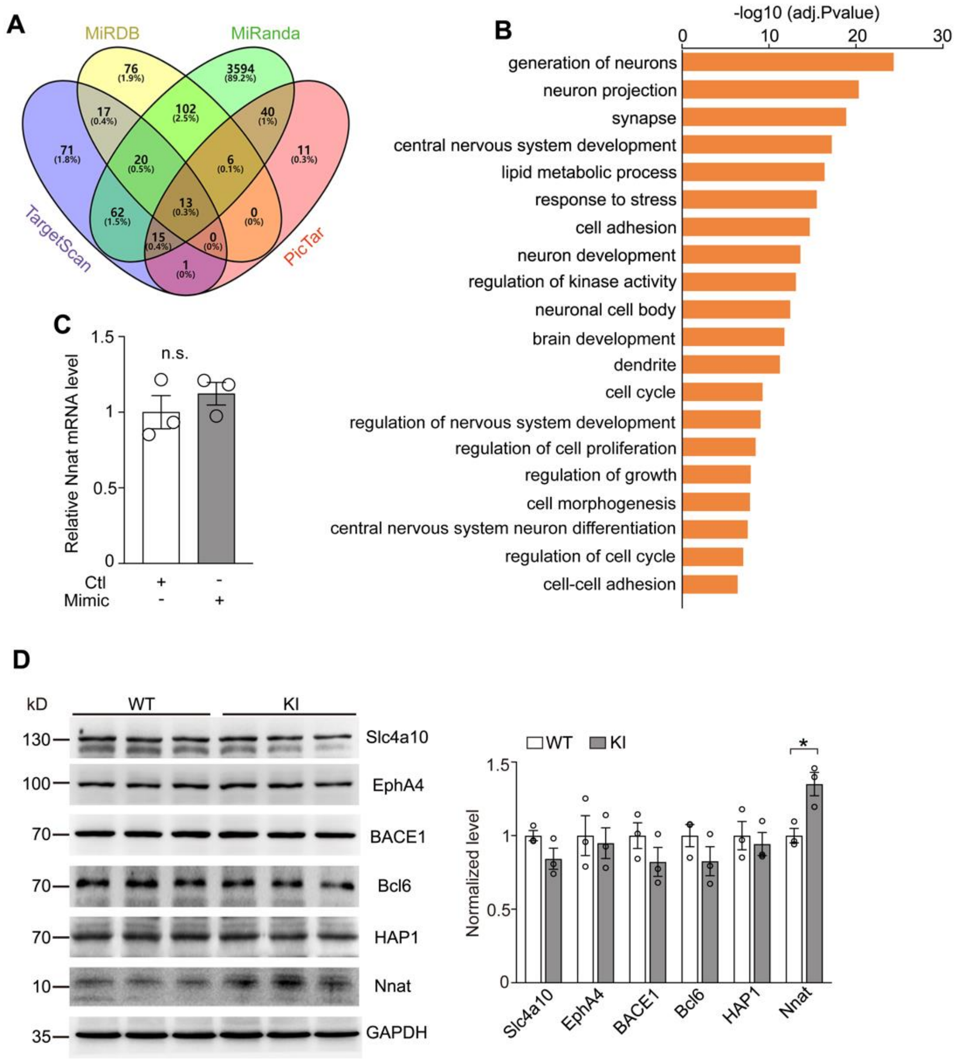

Supplemental Figure 2. Nnat is a novel target of miR-339-5p.

(**A**) Venn diagram of predicted miR-339-5p target genes between TargetScan, PicTar, Miranda and MiRDB databases. 13 candidates are picked up, including *KLFL1*, *PODXl*, *PCYTLA*, *NNAT*, *MYOLC*, *EMILIN2*, *CNOT6*, *BCL6*, *SLC4A10*, *EPHA4*, *HAP1*, *BACE1* and *POMT2*. (**B**) Gene Ontology (GO) analysis of target genes of miR-339-5p. (**C**) Quantification of Nnat mRNA after treating with miR-339-5p mimics or scramble in N2a cells. n=3 independent experiments for each group. (**D**) Immunoblots analysis

and quantification of predicted targets expression in forebrain of 2-month-old WT and KI mice. n=3 for per group. Data are shown as mean ± SEM. $^*p<0.05$ by unpaired t test was used.

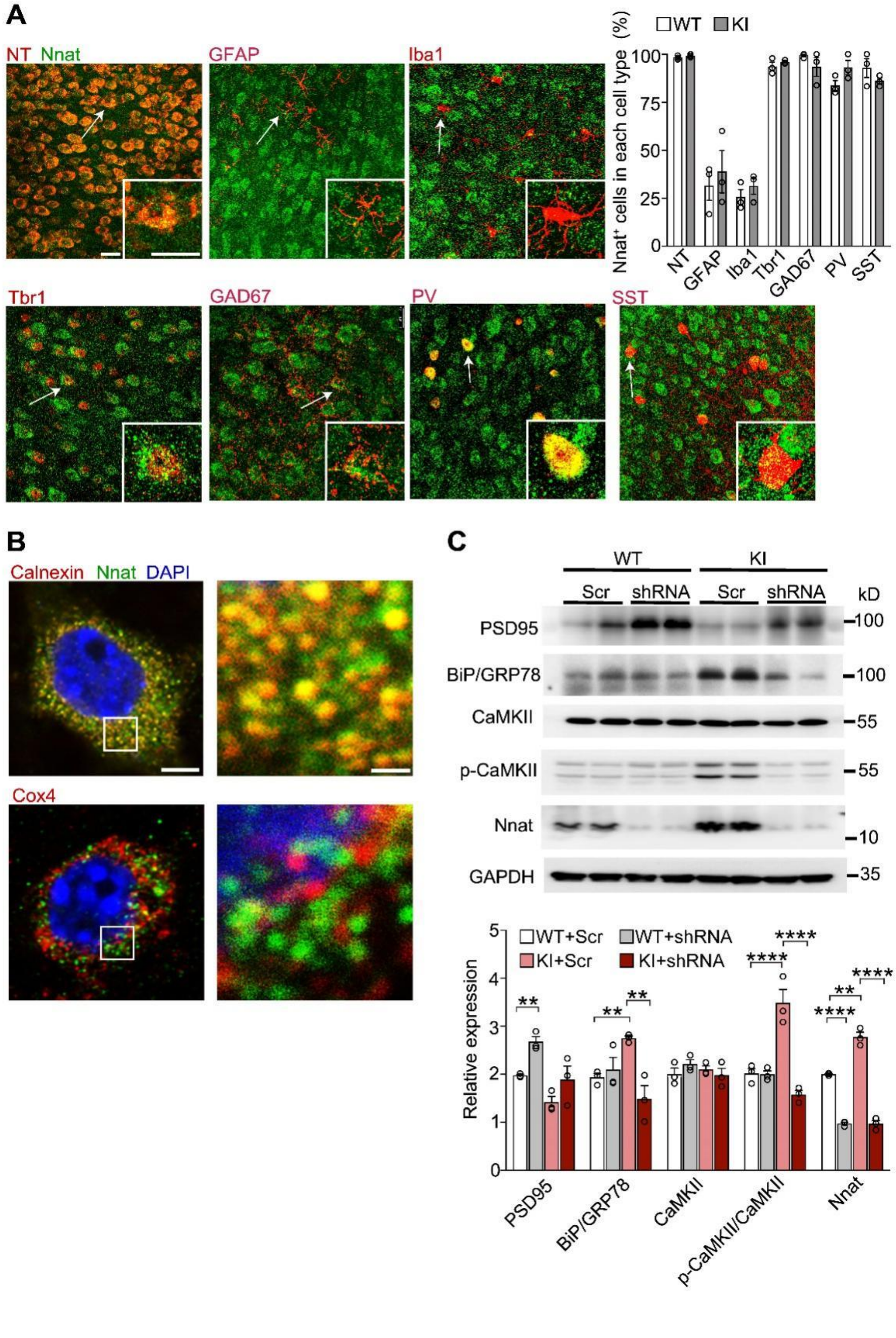

Supplemental Figure 3. Nnat is associated with ER stress

(**A**) Immunohistochemistry staining results showed that Nnat (green) colocalizes with Neurotrace (NT, red), Tbr1 (red), GAD67 (red), parvalbumin (PV, red), and

somatostatin (SST, red), but much less expressed in GFAP (red) positive cells and Iba1 (red) positive cells. White arrows indicate cell marker labeled positive cells. Scale bar: left, 20 µm; right, 5 µm. Lower panel: quantification of the percentage of Nnat positive ($Nnat^{+}$) cell in each cell type in KI and WT mice. n=3 mice for each group. (**B**) Subcellular colocalization of Nnat with calnexin-ER marker and Cox4-mitochondria marker by immunostaining of cortical neurons cultures in vitro. Scale bar: left, 5 µm; right, 1 µm. (**C**) Western blot analysis of different cellular calcium effectors and ER stress proteins after treatment with Nnat shRNA or scramble (Scr) control in WT and KI cultured cortical neurons. n = 3 biological replicates. Values are shown as mean ± SEM. *p<0.05, **p<0.01, ***p<0.001; unpaired t test (**A**) and two-way ANOVA with Tukey's multiple comparison post hoc test (**C**) were used.

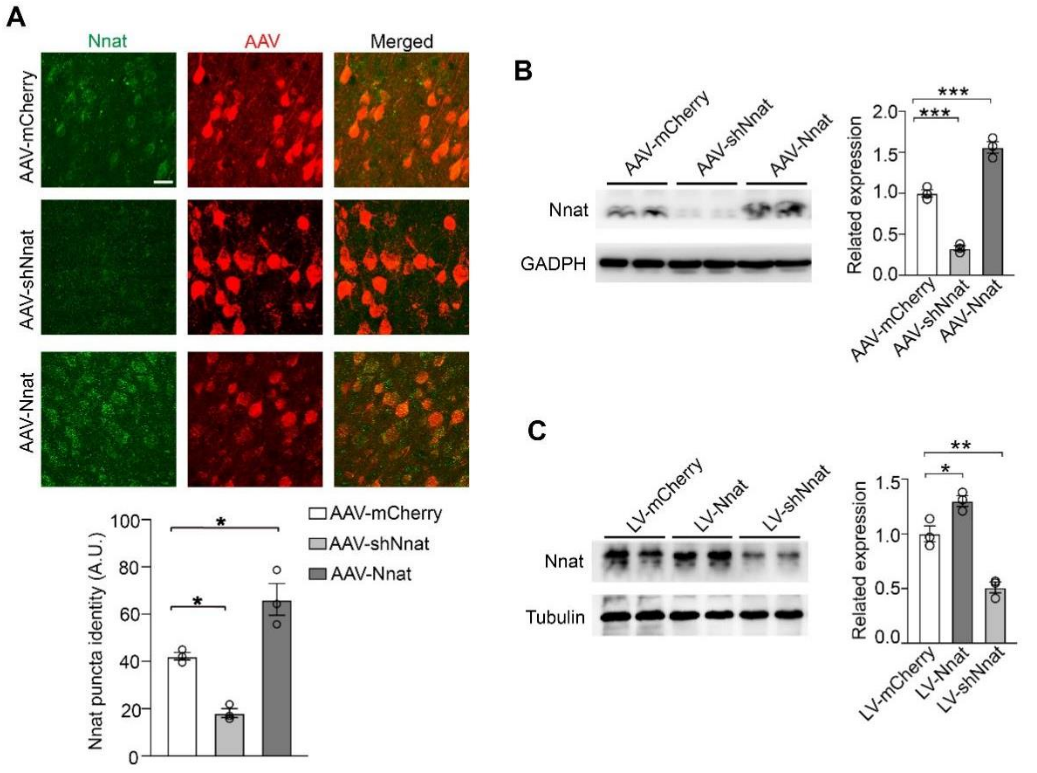

Supplemental Figure 4. The efficiency of Nnat and shNnat AAV and lentivirus.

(**A**) Immunofluorescent staining were used to exam Nnat expression. Lower panel: quantification of Nnat puncta in each group. n=3 for each group. (**B**) Nnat expression in RSC of AAV treated mice were detected by western blot. n = 3 biological replicates. (**C**) Western blot analysis of Nnat expression level in primary neurons were performed after lentivirus transfection. n = 3 biological replicates. Values are shown as mean ± SEM. *p<0.05, **p<0.01, ***p<0.001; one-way ANOVA were used.

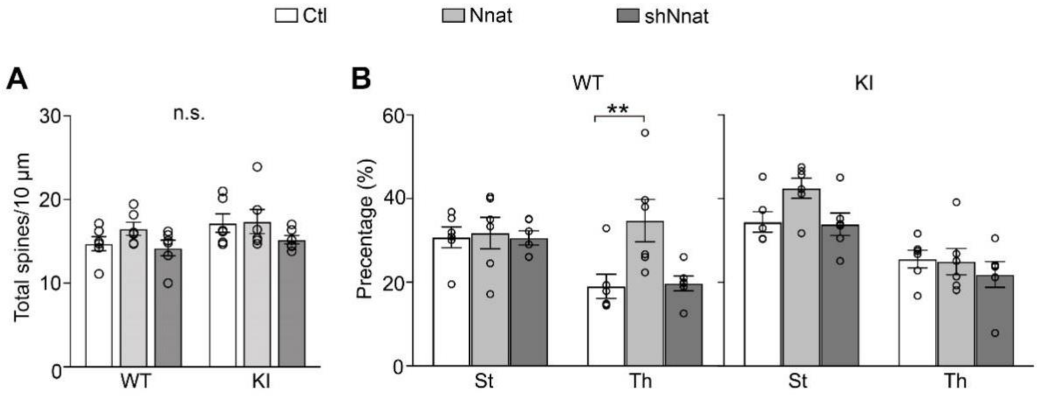

Supplemental Figure 5. Total spines density and stubby type spines show no significant difference in WT and KI neurons after treated with Nnat AAV in vivo. (**A**) Either overexpressing or knocking down Nnat showed no change in total spines density of WT and KI neurons. n=6 mice for each group. (**B**) Nnat overexpression increased thin type spines in WT. n=6 mice for each group. Values are shown as mean ±SEM. ***$p<0.001$; two-way ANOVA with Tukey's multiple comparison post hoc test were used.

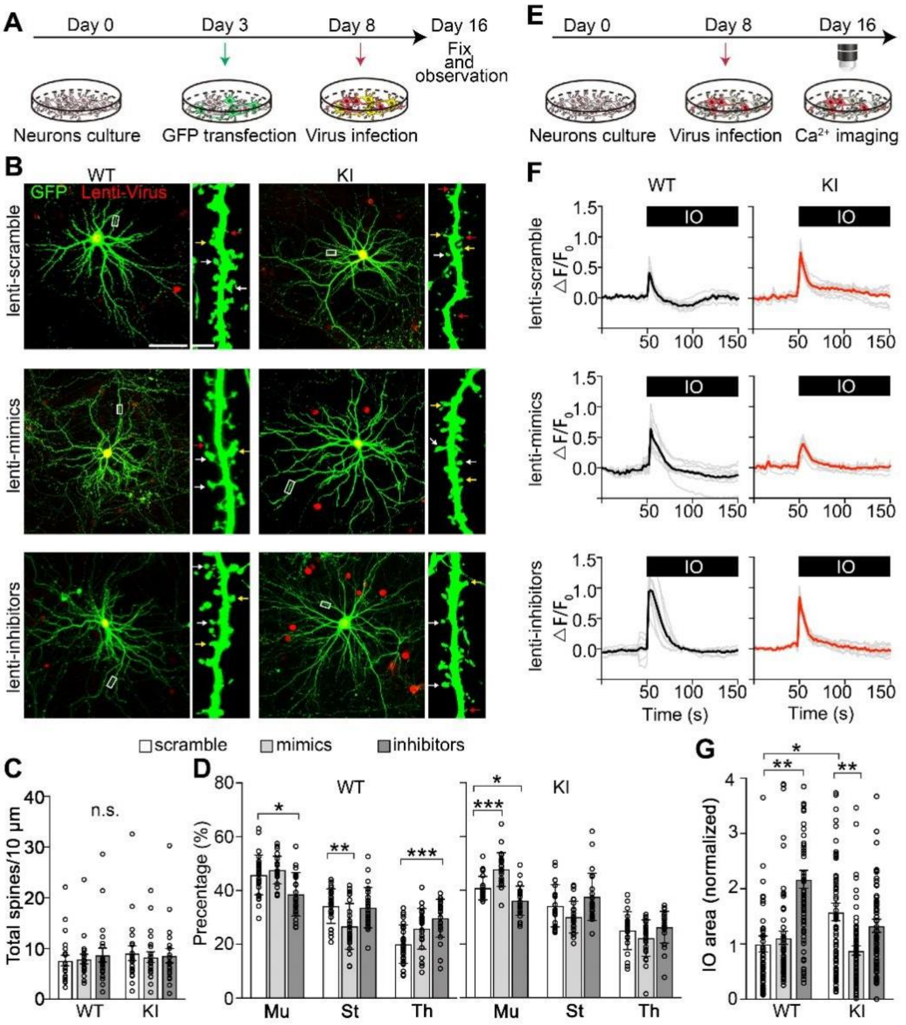

Supplemental Figure 6. MiR-339-5p regulates spine morphogenesis and calcium homeostasis.

(**A**) Schematic of experiments design. After cortical neurons culture, GFP plasmid were transfected on DIV3 and lentivirus were added on DIV. Neurons were ultimately fix and observation on DIV16. (**B**) Representative images of neurons and spines fractions. Scale bars: left, 50 μm; right, 2 μm. Withe arrows indicated mushroom type spines. Yellow arrows indicated stubby type spines and the red arrows indicated thin type spines. (**C**) Total spines density of WT and KI cultured neurons treated with miR-339-5p control, mimics or inhibitors respectively. n=20~21 neurons for each group. (**D**)

Quantification of number of mushroom (Mu) type, stubby (St) type and thin (Th) type. 3~5 dendrites per neuron were calculated. n=23~25 neurons for each group. (**E**) Schematic of experiments design. After cortical neurons culture, lentiviruses were added on DIV 8 and calcium imaging experiments were performed on DIV16. (**F**) Time-course of Fura-2 Ca^{2+} signals (F340/F380) in the ER of neurons from WT and KI mice infected with lentivirus individual. Cell trace (grey) and average trace (black for WT and red for KI) are shown for each group. (**G**) Quantification for average sizes of ionomycin (IO) induced Ca^{2+} estimated as area under curve (AUC) of Fura-2 signal in each group responses in soma of WT and KI cortical neurons treated with different lentivirus (normalized to control group). n= 60~79 neurons. Values are shown as mean ± SEM (All the data was collected from 3 batches of cultures). *p<0.05, **p<0.01and ***p<0.001by two-way ANOVA of Turkey's test.

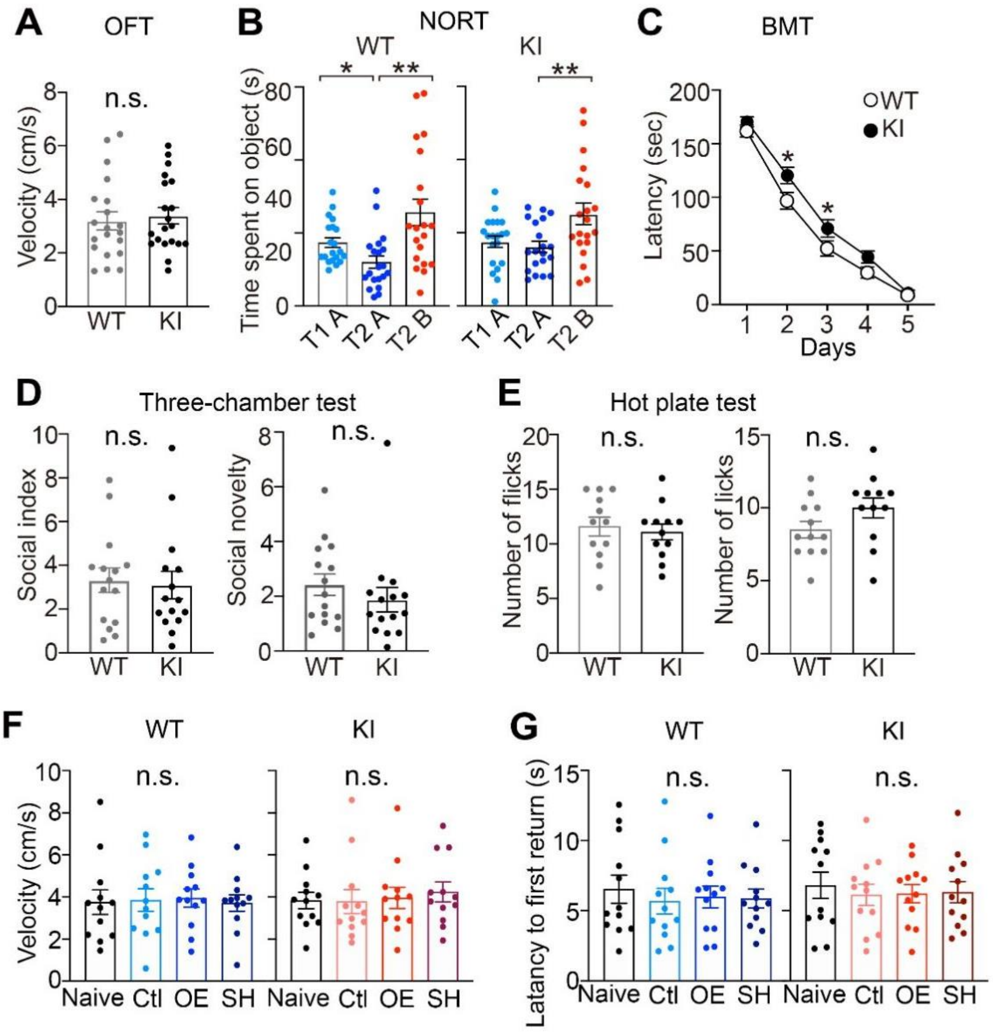

Supplemental Figure 7. Two-month-old KI mice show mild learning defect.

(**A**) Average velocity of WT and KI mice at 2 months on the open field test. n=20 mice for each group. (**B)** Accumulation time spent on different objects on the short-term novel objects recognition test. n=20 mice for each group. (**C**) On the Barnes maze test (BMT), latency to hiding holes were recorded. n=20 mice for each group. (**D**) Social behavior in three-chamber test. Sociality index (time interacting with Stranger 1/time interacting with Empty) and social novelty (time spent in Stranger 1 /time spent in Stranger 2) of WT and KI mice were calculated, respectively (right panel). n = 15 mice for per group. (**E**) Hot-plate test. The number of flicks (left) and licks (right) were

calculated of WT and KI mice at 2 months. (**F**) Average velocities were recorded of the WT and KI mice. n=15 mice for per group. (**G**) Latency to first return were recorded to assess the vision function. n=12 mice for per group. Values are shown as mean ± SEM. One-way ANOVA was used. Data are shown as mean ± SEM. *p<0.05, **p<0.01 by two-way ANOVA (**C**, **F**, **G**) or unpaired t-test (**A**, **B**, **D**, **E**).

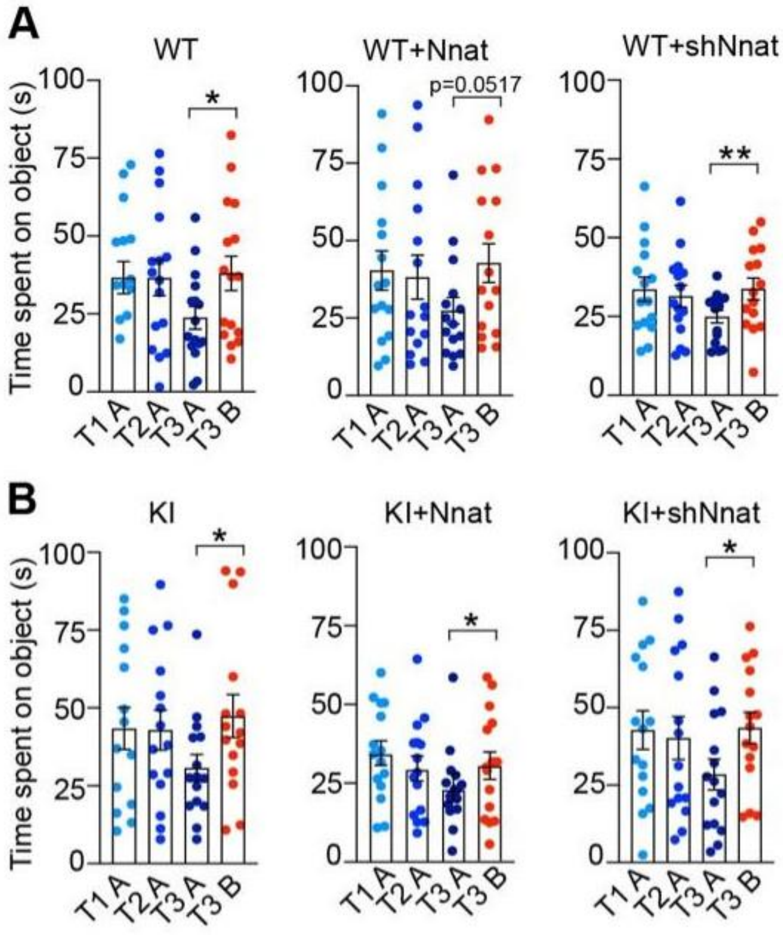

Supplemental Figure 8. Behavior defect do not show in the consecutive three days novel object recognition test.

(**A**) The time spent on different objects were calculated for WT control group, Nnat overexpression in WT group, and AAV-shNnat injected WT mice. n=15 mice for each group. (**B**) The time spent on different objects were calculated for KI control group, Nnat overexpression in KI group, and AAV-shNnat injected KI mice. n=15 mice for each group. Data are shown as mean ± SEM. *$p<0.05$ by one-way ANOVA.

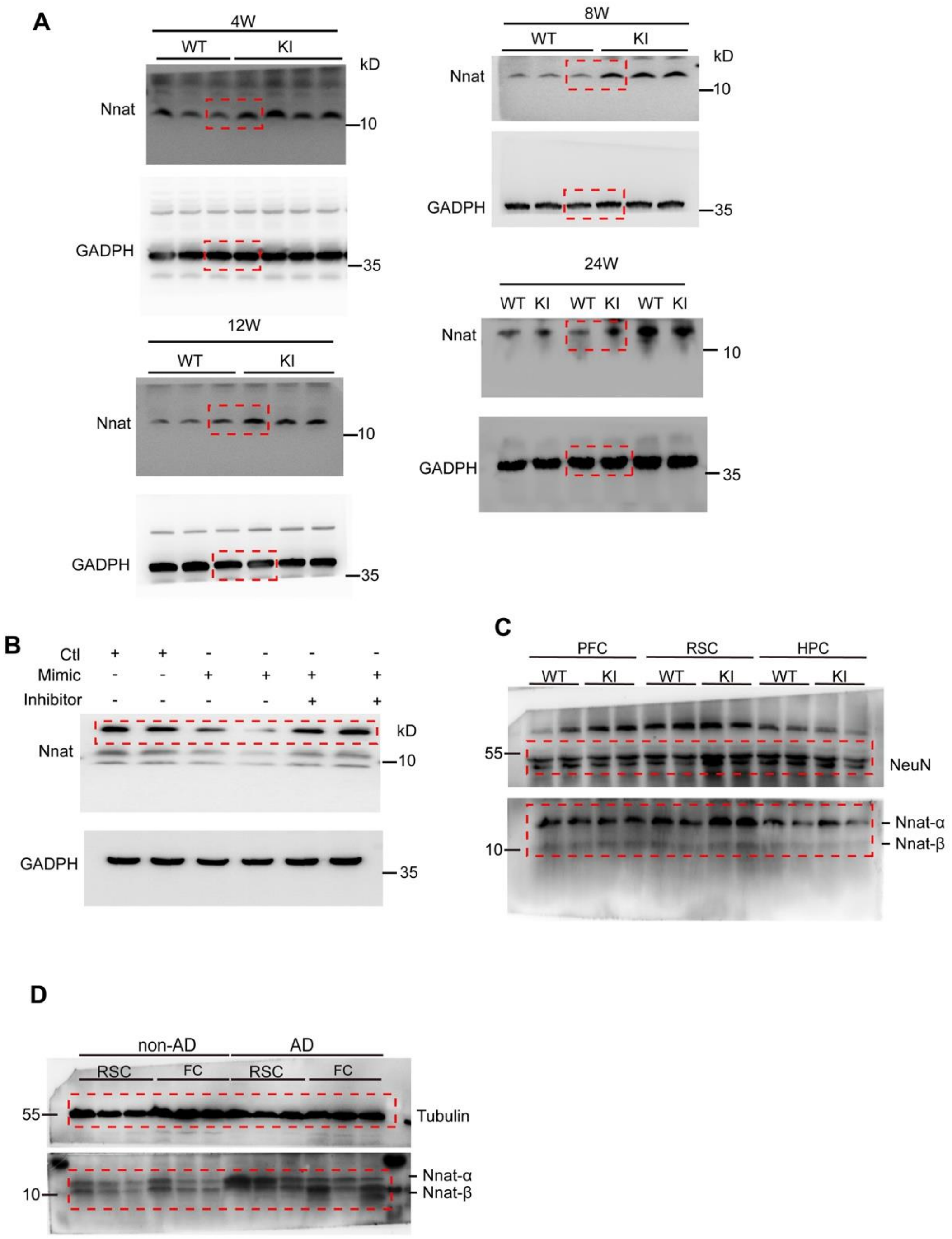

Uncut gels 1. The whole gel images for western blot results in Figure 1 and Figure 2.

(**A**) The whole gel imags for Figure 1F. (**B**) The whole gel imags for Figure 1E. (**C**)The whole gel imags for Figure 2B. (**D**) The whole gel imags for Figure 2C. Red dotted lines indicated the part showed in Figures.

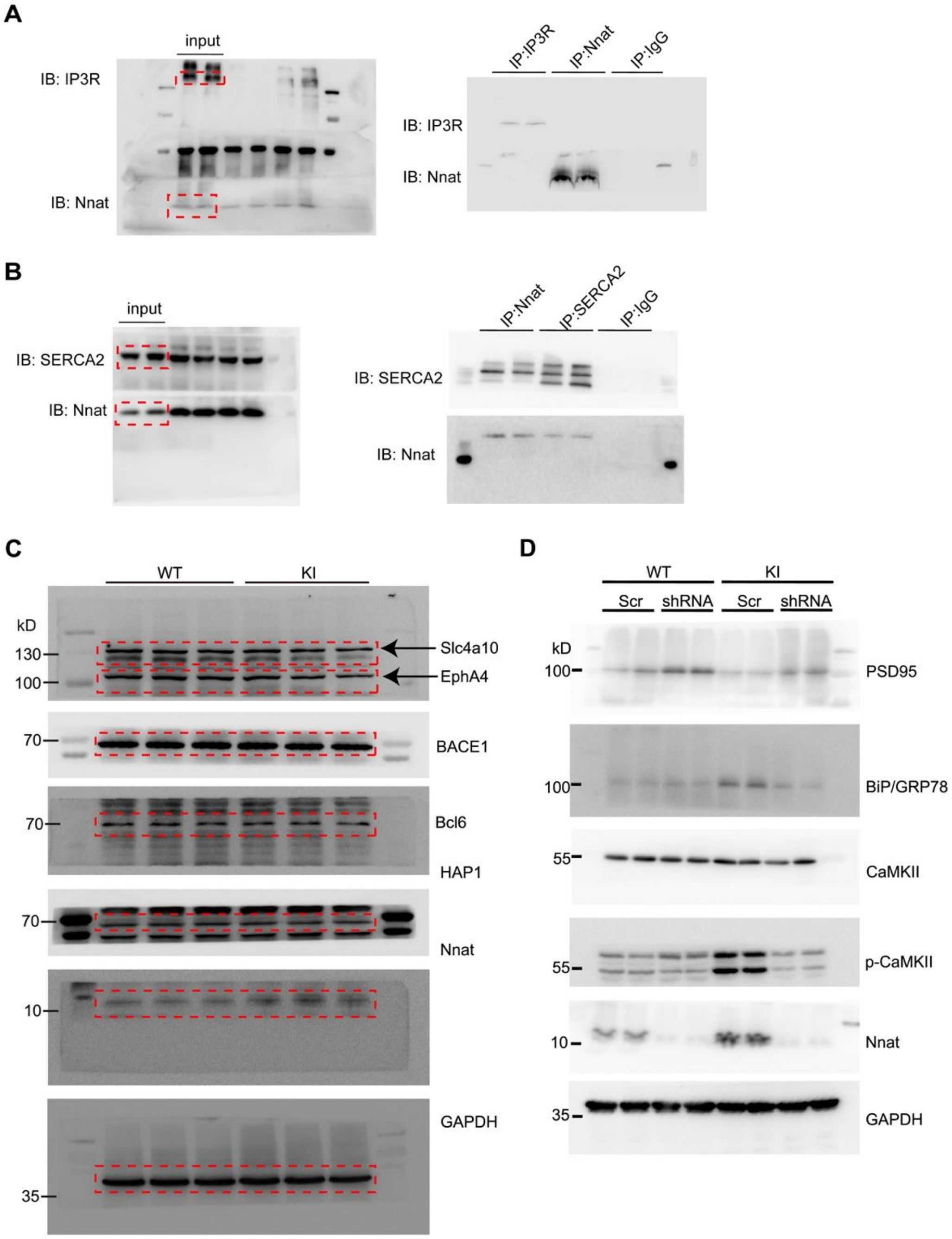

Uncut gels 2. The whole gel images for western blot results in Figure 4, Supplemental Figure 2 and Supplemental 3.

(**A**) The whole gel images for western blot result in Figure 4C. (**B**) The whole gel images for western blot result in Figure 4D. (**C**) The whole gel images for western blot result in Supplemental Figure 2D. (**D**) The whole gel images for western blot result in Supplemental Figure 3C. Red dotted lines indicated the part showed in Figures.
